# Use of a Discrete Choice Experiment to Inform De-implementation of Mammography Overscreening: A US-Based National Survey

**DOI:** 10.1007/s11606-025-10158-9

**Published:** 2026-01-27

**Authors:** Nathalie Moise, Dallas Wood, Jennifer Mizhquiri Barbecho, Anita G. Karr, Savannah P. Alexander, Rachel C. Shelton, Parisa Tehranifar

**Affiliations:** 1 Columbia University Irving Medical Center, New York, NY USA; 2 Western Carolina University, Culowhee, NC USA; 3 Center for Applied Economics and Strategy, RTI International, Research Triangle Park, Durham, NC USA; 4 Department of Medicine, Columbia University Irving Medical Center, New York, NY USA; 5 Department of Epidemiology, Mailman School of Public Health, Columbia University, New York, NY USA; 6 Department of Sociomedical Sciences, Mailman School of Public Health, Columbia University, New York, NY USA; 7 Irving Institute for Clinical and Translational Research, Columbia University, Medical Center, New York, NY USA; 8 Herbert Irving Comprehensive Cancer Center, Columbia University, Medical Center, New York, NY USA

**Keywords:** mammography, older women, de-implementation science, discrete choice experiments, overscreening, decision making, patient preference

## Abstract

**Background:**

Mammography overscreening, defined as any routine screening in women ≥ 75 years, particularly with limited life expectancy, persists.

**Objective:**

Identify preferences for de-implementing mammography overscreening among older women.

**Design:**

A national survey using the NORC AmeriSpeak panel, a probability-based panel representative of US households. Informed by qualitative methods, we constructed a discrete choice experiment (DCE) based on a hypothetical patient activation de-implementation strategy (the Rethink Resource) for prompting patient/provider discussions about whether to stop getting mammograms.

**Participants:**

Women ≥ 70 years old selected using sampling strata based on age, race/ethnicity, and education and without a breast cancer history.

**Main Measures:**

Attributes (levels) included modality (electronic, paper, in-person); context (reviewed with provider, group, on their own); content (mammography pros/cons, patient story/testimonial); frequency (once, yearly); and decision-making principles (age/health calculator, personal preferences/responsibilities checklist). We estimated a random utility model to quantify patient preferences and calculate importance scores.

**Results:**

There were 673 eligible participants; the weighted mean age was 77.5 (standard deviation: 5.3); 72.0% were Non-Hispanic White, 10.5% Non-Hispanic Black, and 9.8% Hispanic; 69.3% had less than a college degree; 49.6% agreed with the idea of stopping mammography based on age and health. In order of importance, participants preferred (mean [standard error]) the Rethink Resource be reviewed: with their healthcare provider (1.52 [0.08]) or on their own (1.22 [0.07]), include pros/cons (0.79 [0.05]), and be delivered on paper (0.81 [0.07]) or electronically (0.60 [0.07]) on a yearly basis (0.34 [0.05]). There were no significant preferences for decision-making principles (–0.01 [0.05]).

**Conclusions:**

In the first DCE for de-implementation strategies, we found that women express clear preferences for how and with whom information is relayed, but do not have strong preferences for calculators/checklists.

**Supplementary Information:**

The online version contains supplementary material available at 10.1007/s11606-025-10158-9.

## INTRODUCTION

Current guidelines do not recommend routine mammography in average-breast-cancer-risk women ≥ 75 years old, citing insufficient evidence to balance the harms and benefits^1^ and recommending consideration of health status, life expectancy, and patient preferences in determining whether to continue or discontinue mammography.^2–4^ Potential harms of overscreening include unnecessary testing, psychological distress due to false positives, physical pain, and costs^5,6^ without clear reduction in mortality.^7^ Overscreening has been defined as routine screening among patients older than the guideline recommended upper age limit, with limited life expectancy or at a greater frequency;^8^ one study estimated that 56.2% of women are overscreened (i.e., defined as any screening ≥ 75 years) for breast cancer.^9^ The emerging field of de-implementation science^10,11^ —the study of systematically “reducing (frequency and/or intensity) or stopping the use or delivery of health services or practices that are ineffective, unproven, harmful, overused, inappropriate, and/or low-value by practitioners and delivery systems to patients” — has the potential to inform how best to reduce mammography overscreening. Research to date has focused on qualitatively elucidating older women’s mammography screening attitudes (which are favorable and associated with strong emotions),^12–14^ or, to a lesser extent, testing the impact of decision aids on mammography screening in older women.^12^ It may be appropriate to continue mammograms in patients with a life expectancy of ≥ 10 years and decision-making should be individualized. Decision aids/shared decision-making align with implementation strategy taxonomy (i.e., prepare patients and consumers to be active participants)^15^ but are rarely theory-driven/selected to target determinants of overscreening. As such when decision aids are used, more than 40% of women with a life expectancy of < 10 years continue mammography.^16^ Limited progress has also been attributed to action bias (i.e., disbelief that cancer screening might not save lives), cancer worry, and varying reassurance.^17^ Nonetheless, women are rarely involved in designing de-implementation efforts.

A discrete choice experiment (DCE) (also known as conjoint analysis) is a method for eliciting and quantifying preferences for various options of a product as well as the potential trade-offs individuals are willing to make.^18,19^ Implementation scientists increasingly highlight the utility of DCEs for engaging stakeholders and selecting *implementation* strategies.^20,21^ While DCEs have been used to elucidate preferences for low evidence therapies (e.g., x-rays for back pain)^22^ and drivers of treatment decision-making (e.g., patient characteristics, treatment options),^23^ they have rarely been applied to design de-implementation strategies themselves. Unique design and methodological considerations remain unknown.

We aimed to conduct a DCE in a nationally representative sample of older women to understand preferences for a patient activation strategy for reducing mammography overscreening.

## METHODS

### Overview

To finalize the content and delivery of a patient-level de-implementation strategy (The Rethink Resource, a patient activation guide that prompts discussions between patients and their providers), we deployed a DCE in a nationally representative sample of US women ≥ 70 years old with no breast cancer history, using the National Opinion Research Center (NORC) AmeriSpeak® panel. Participants provide informed consent to receive ongoing surveys when they join the AmeriSpeak panel. Members can choose whether to complete subsequent surveys or not. For every client study, AmeriSpeak submits a waiver for documentation of consent. The NORC IRB determined this survey to be exempt under federal regulations. The Columbia University Institutional Review Board (IRB) approved this study (AAAQ1705). The study followed the American Association for Public Opinion Research (AAPOR) and the Strengthening the Reporting of Observational Studies in Epidemiology (STROBE) reporting guidelines.

### Survey Design

Our DCE approach was informed by the International Society for Pharmacoeconomics and Outcomes Research (ISPOR) checklist for good research practices.^18^ We previously conducted an innovation tournament, a participatory design approach for crowdsourcing implementation system/provider-level ideas among radiologists, primary care clinicians, community health workers/educators, and specialists (e.g., oncologists) to select strategies for reducing mammography over-screening.^24^ The highest rated strategies included healthcare system alignment on guidelines (e.g., consensus on reminder letter language); integrating mammography guideline aligned electronic health record features; and clinician education/training; as well as patient-level shared decision-making tools and education to promote patient activation. To incorporate patient input and better select, refine, and tailor this patient activation strategy, our research team, advisory board, and a creative director iteratively conducted rapid prototyping of both our Rethink Resource and CDCE to identify important Resource components.^25^

To arrive at our final set of DCE attributes and levels, we (1) reviewed our previously conducted qualitative studies with patients and providers on barriers/facilitators to reducing mammography overscreening to identify needs of older patients^13,14^; (2) conducted one-on-one “strategy refinement” interviews with 17 women (mean age 80.1 [SD = 3.77], 7 Spanish-speaking, 10 English) from the Columbia University Irving Medical Center catchment area (i.e., mammography clinic, primary care referrals, and community flyers) to identify unique de-implementation needs and range of Resource preferences; and (3) iteratively reviewed versions of the DCE survey with our advisory board of mammography, geriatrics, and implementation science experts (*n* = 11). Based on feedback, we created a (1) carefully constructed description of the guidelines and question for eliciting guideline acceptability; (2) brief video describing a tangible patient activation strategy (the “Rethink Resource”) for prompting provider/patient discussions; and (3) guide/example for selecting options in a DCE. To simplify the DCE in our older population, some of whom it would be reasonable to continue mammogram (e.g., those ≥ 10-year life expectancy) and/or would not endorse stopping mammography, we arrived at a final DCE question stem that asked patients to select the option that would prompt them to *have a discussion with their provider* about whether to stop getting mammograms (vs. which of these options would prompt you to stop getting mammograms). Given participant age, we also limited the number of attributes/levels and focused on areas where there were uncertainty and lack of consensus, and would result in actionable resource refinement. For example, advisory board experts felt it was important to differentate content from *how* patients preferred to make the final decision (i.e., decision-making principles), which could be via a general consideration of preferences/life commitments/values or with the use of age/health-based calculators. Attribute/levels were not meant to be exclusive (i.e., content could eventually include both stories/testimonials and facts) but to elucide which was most important to patients (Supplementary Material 2).

### Procedures

From May 2024 to July 2024, we fielded a survey using AmeriSpeak®, a probability-based panel funded and operated by NORC, designed to be representative of US households.^26^ The eligibility criteria (e.g., age ≥ 70 years) were informed by qualitative data with patients, clinicians, administrators^13,14^, and input from our multi-stakeholder advisory board. The sample was selected from the panel using sampling strata based on age, race/Hispanic ethnicity, education, and gender (18 sampling strata in total, see Supplementary Material 1). The size of the selected sample per stratum was determined such that the distribution of the complete surveys across the strata matched that of the target population as represented by census data. We selected a sample of women ≥ 70 years old using strata based on age (70–74, 75–79, 80+), race/ethnicity, and education, attempting to oversample Hispanic, Black, and “other” race participants.

Participants were invited via email and conducted a 12–15-min online questionnaire in English. The survey (Supplementary Material 3,Page 30–39) elicited (1) demographics, personal/family history of breast cancer history, prior mammograms, and the PROMIS general health status single-item question^27^; (2) whether/when the participant was planning on getting a mammogram; (3) whether a healthcare provider informed participants regarding choice to get a mammogram^28^; (4) preferred decision-maker for whether to stop mammograms (patient, family member, and/or healthcare provider); and (5) agreement with the idea of stopping mammograms based on both age and health (while potentially distinct aligned with stakeholder preferences and the majority of guidelines).^2–4^

**Figure 1:**
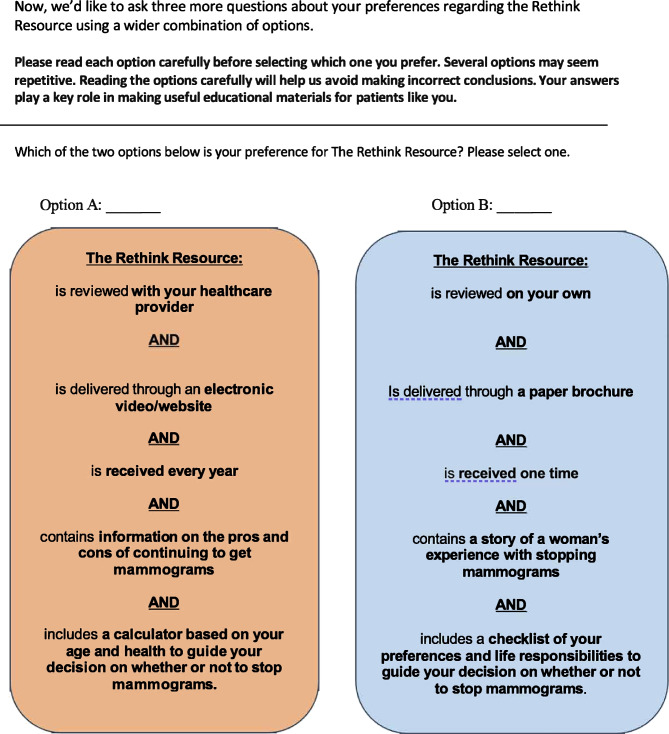
Example of options in the discrete choice experiment. This figure represents an example of 2 options in a full profile (5x5) DCE. Partial profiles included just 2x2 options.

The DCE involved five attributes of the patient activation de-implementation strategy (“The Rethink Resource”) to prompt discussions with healthcare providers about whether to stop getting mammograms: (a) modality (electronic, paper, in-person, phone); (b) context (reviewed with healthcare provider, group of women (e.g., YWCA), on their own); (c) content (information on pros/cons of mammograms, patient story/testimonial about stopping mammograms); (d) frequency (once, yearly); and (e) decision-making principles (a calculator based on your age and health vs. a checklist of your preferences and life responsibilities to guide your decision on whether or not to stop mammograms). Participants received 15 choice questions: 12 “partial profile” choice questions where participants considered two attributes at a time and three full profile questions with all attributes (Fig. 1). Based on Orme rule of thumb (number of attributes, levels, etc.), we determined that we would need at least 300 participants.^29^ To assess the internal validity and quality of data collected from the DCE, we administered three internal validity evaluations, including attention checks, questions assessing ease and understanding,^19^ and straightlining (i.e., whether respondents selected the same option in each DCE question)^30^ (Supplementary Material 3, page 2).

### Analyses

We conducted analyses using survey weights prepared by NORC to obtain nationally representative estimates. Descriptive statistics were calculated for attention checks as well as the frequency, and unweighted and weighted proportions of demographic variables and attitudes.

We estimated *random utility models (RUMs)* that quantified patient preferences for each resource attribute in the DCE. We estimated a RUM for each question type for all questions (partial and full profiles). To estimate the RUM, we assumed that each participant selected the resource option that provides the highest level of utility. We defined the RUM as: *U*_ijk_ = *β*_REVIEW1_ × REVIEW1_ijk_ + *β*_REVIEW2_ × REVIEW2_ijk_ + *β*_MODALITY1_ × MODALITY1_ijk_ + *β*_MODALITY2_ × MODALITY2_ijk_ + *β*_FREQ1_ × FREQ1_ijk_ + *β*_CONTENT1_ × CONTENT1 + *β*_PRINC1_ × PRINC1_ijk_; where *U* is the *i*th individual’s utility of choosing option *k* in question *j*. For example, *REVIEW1*_*ijk*_ equals 1 if option *k* in question *j* says the Rethink Resource is reviewed with your healthcare provide and equals 0 if not; *REVIEW2*_*ijk*_ equals 1 if option *k* in question *j* says the Rethink Resource is reviewed on your own and equals 0 if not (See Supplementary Material 2).

The coefficients of the RUM above are often called “preference weights.” These coefficients reflect how much satisfaction a participant receives from attribute level over the excluded attribute level. We also converted all preference weights to odds ratios (ORs) by exponentiation of the RUM coefficients. ORs corresponded to the odds of an individual preferring a hypothetical Rethink Resource with a given design attribute versus an alternative hypothetical Rethink Resource with a different design attribute. We estimated all preference weights using conditional logit regression.^31^

As a final step, we used the coefficient estimates from each RUM to calculate an importance score for each attribute (i.e., the difference between the most preferred level and the least preferred level of each attribute) using the estimated preference weights. We added these differences and calculated what proportion of the sum was associated with each attribute.^32^ The standard errors and the 95% confidence interval (CI) for these importance scores were calculated using the delta method, a statistical technique that estimates the variance of a nonlinear combination of random variables (such as importance scores) using a first-order Taylor-series expansion (Supplementary Material 3, pages 2–4).^33^

### Sensitivity Analyses

We conducted three sensitivity analyses (Supplementary Material 3, pages 3–4). First, we examined all survey outcomes in the total sample of individuals who completed the survey (inclusive of those with a history of breast cancer). Second, we investigated whether our results were sensitive to the choice of estimator by estimating the preference weights using random-parameters logit (RPL) instead of conditional logit.^34–38^ The third sensitivity analysis explored differences in preferences between partial and full profile questions.

We performed all analyses in Stata 18.0 (StataCorp, College Station, Texas). We provide technical details for the AmeriSpeak® panel in Supplemental Material 1 and DCE survey and methods, including attention and internal validity checks and survey, in Supplemental Material 3.

## RESULTS

### Participant Characteristics and Attitudes

Of 2575 participants approached, 819 completed the survey (31.8%). We excluded 146 (17.8%) for prior breast cancer diagnosis, leaving 673 eligible participants. In weighted results, the mean age was 77.3 (5.3); 72.0% were Non-Hispanic (NH) White, 10.5% NH Black, and 9.8% Hispanic; 69.3% had less than a college degree; 62.7% made < $60,000 annually; 24.0% had a first-degree relative with breast cancer; and 46.2% reported excellent/very good health. In all, 70.8% were planning to get a mammogram every 1–2 years, and 38.8% had been told by their healthcare providers that mammography is a choice (Table 1). Once presented with a brief summary of guidelines in older women, 49.6% agreed with the idea of stopping screening mammography based on age and health.

**Table 1:** Demographics and Mammography Screening Preferences Among Women ≥ 70 Years Old (*n*=673)

Characteristics	Number of participants	Unweighted percentage	Weighted percentage
**Age **			
70–74 years old	161	23.9	34.0
75–79 years old	421	62.6	31.0
80 years old or older	91	13.5	35.0
**Race/ethnicity**			
White, non-Hispanic	493	73.3	72.0
Black, non-Hispanic	100	14.9	10.5
Other non-Hispanic (Asian, 2+ races, etc.)	33	4.9	7.7
Hispanic	47	7.0	9.8
**Education**			
Less than high school	7	1.0	1.3
High school or equivalent (or GED)	103	15.3	26.0
Some college or associate’s degree (e.g., AA, AS)	253	37.6	42.0
Bachelor’s degree (e.g., BA, BS)	152	22.6	15.6
Post-graduate study or professional degree	158	23.5	15.1
**Marital status**			
Married	306	45.5	41.2
Not married	367	54.5	58.8
**household income**			
Less than $30,000	139	20.7	22.1
$30,000 to under $60,000	250	37.2	40.6
$60,000 to under $100,000	154	22.9	23.0
$100,000 or more	130	19.3	14.3
**Region**			
Northeast	92	13.7	17.0
Midwest	160	23.8	20.7
South	223	33.1	38.7
West	198	29.4	23.7
**Would you say your health in general is?**			
Excellent	49	7.3	6.9
Very good	274	40.7	39.3
Good	258	38.3	39.9
Fair	78	11.6	12.1
Poor	11	1.6	1.5
Skipped	3	0.5	0.3
**First-degree biological relative diagnosed with breast cancer**			
Yes	151	22.4	24.0
No	509	75.6	71.8
Not sure	12	1.8	4.1
Skipped	1	0.2	0.1
**Last mammogram **			
A year ago or less	417	62.0	56.7
More than 1 year, up to 2 years ago	100	14.9	14.5
More than 2 years, up to 3 years ago	47	7.0	7.8
More than 3 years ago	92	13.7	18.9
I’ve never had a mammogram	16	2.4	2.1
Skipped	1	0.2	0.1
**Mammogram planning**			
Plan to get a mammogram every 1–2 years or so	512	76.1	70.8
Don’t plan to get mammogram in next few years	40	5.9	8.2
I’ve decided to stop altogether	65	9.7	13.0
I’m undecided/other	56	8.3	8.0
**Did a healthcare provider tell you that you could choose whether or not to have a mammogram?**			
Yes	251	37.3	38.8
No	420	62.4	60.9
Skipped	2	0.3	0.4
**Agreement with idea of stopping mammograms based on a woman’s age and health**			
Strongly/somewhat agree	316	47.0	49.6
Neutral	139	20.7	20.9
Somewhat/strongly disagree	218	32.4	29.5

### Results of Internal Validity Checks

Overall, three (0.4%) participants failed the attention test. There was evidence of straightlining in 2.2% of all choice questions, with evidence of more straightlining among the full profile questions (26.9%). For both partial and full profile DCE questions, the vast majority of participants found the questions easy to understand and answer (≥ 72%) (Supplementary Material 3, pages 7–8).

### Results of Discrete Choice Experiment

**Figure 2:**
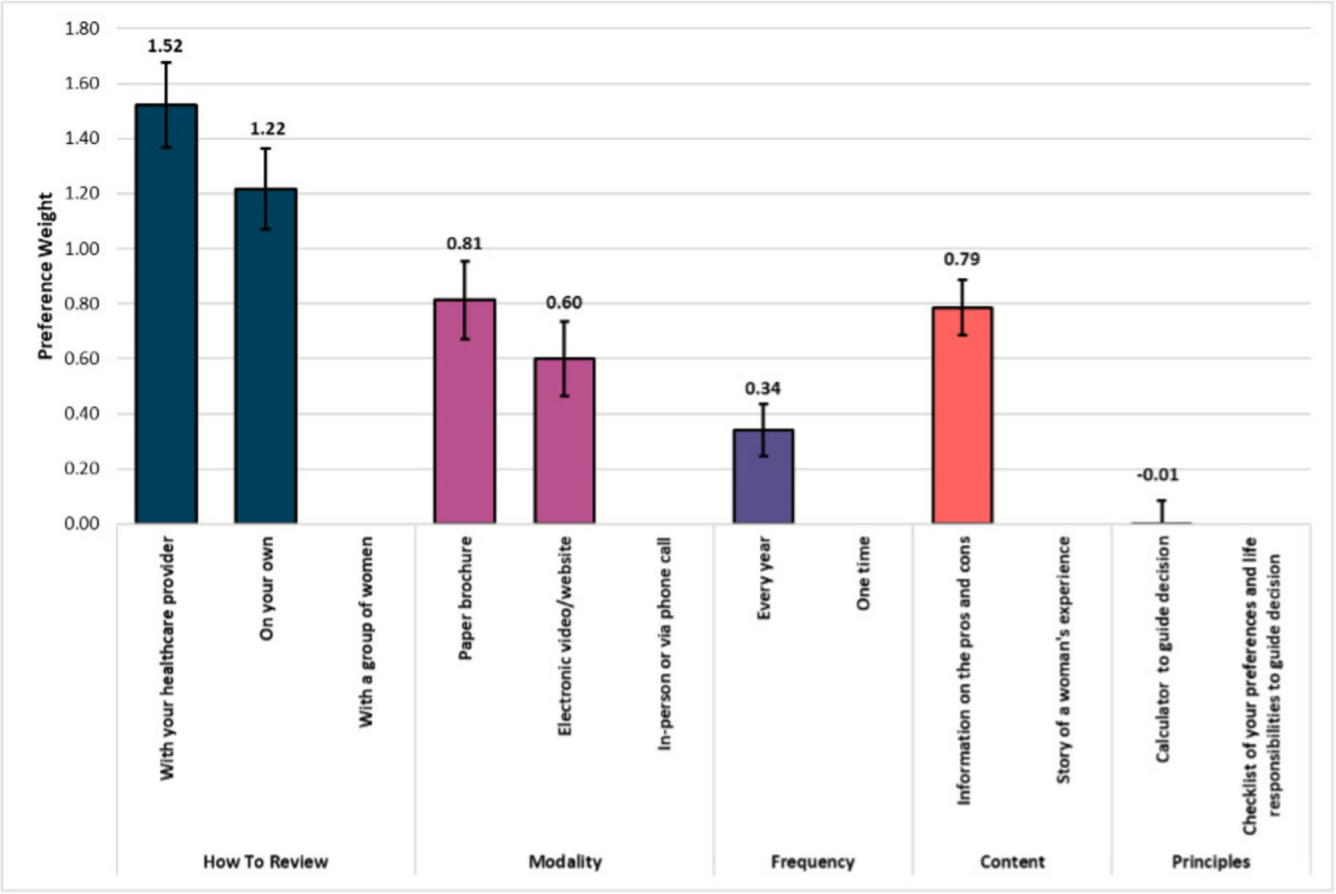
Conditional logit preference weights (all DCE questions ). This figure represents the preference weights estimated for random utility model using all DCE questions.

Participants preferred (mean [standard error]) the Rethink Resource be reviewed with their healthcare provider (1.52 [0.08]) or on their own (1.22 [0.07]) (vs. in a group) and that it include facts on pros/cons (vs. a patient story/testimonial) (0.79 [0.05]). In terms of modality, participants preferred that the resource be delivered on paper (0.81 [0.07]) or electronically (0.60 [0.07]) (vs. reviewed in real time with a person/phone). They preferred to receive the Rethink Resource on a yearly basis (vs. once) (0.34 [0.05]). We found no significant preferences for incorporation of decision-making principles (−0.01 [0.05]) (Table 2, Fig. 2).

**Table 2:** Conditional Logit Results (Weighted) of the Discrete Choice Experiment (all DCE questions)

	All DCE questions	
	Mean (Std. Err.)	Odds ratio
**How to review the information**		
Reviewed with your healthcare provider vs. reviewed with a group of women	1.52*†(0.08)	4.6
Reviewed on your own vs. reviewed with a group of women	1.22*†(0.07)	3.4
**Modality **		
Delivered through a paper brochure vs. delivered in person or via phone call	0.81*†(0.07)	2.3
Delivered through an electronic video/website vs. delivered in person or via phone call	0.60*†(0.07)	1.8
**Frequency**		
Received every year vs. received one time	0.34*(0.05)	1.4
**Content **		
Information on the pros and cons of stopping mammograms vs. a story/testimonial of a woman’s experience with stopping mammograms	0.79*(0.05)	2.2
**Decision-making principles **		
Calculator based on your age and health vs. checklist of your preferences and life responsibilities	−0.01(0.05)	1.0

Source: 2024 De-implementation of Mammography Survey, *N* = 673. Standard errors are in parentheses

*The coefficient is statistically different from 0 at the 5% significant level

†Two coefficients for the same attribute are statistically different from each other at the 5% significance level

**Figure 3:**
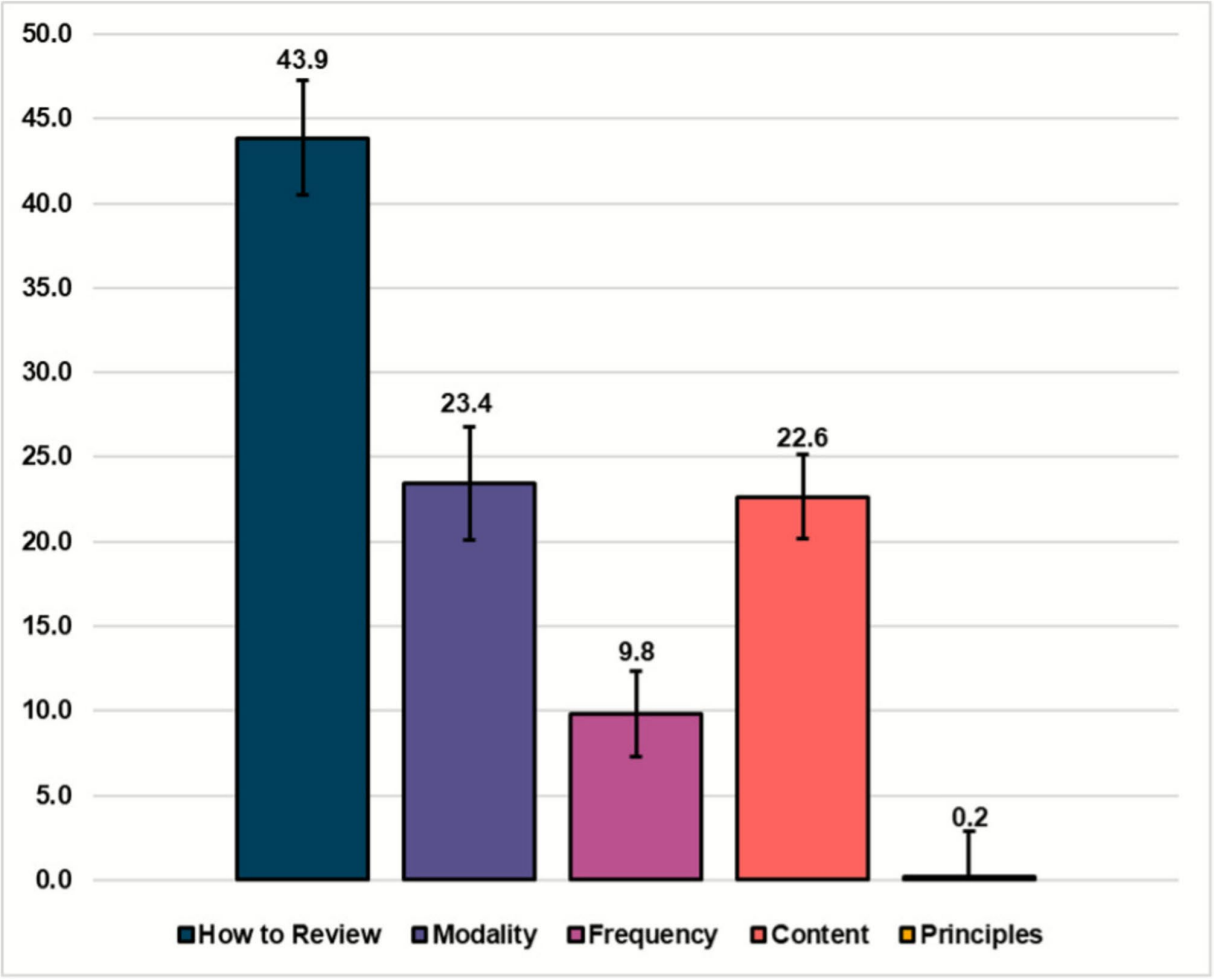
Attribute importance score estimated using conditional logit. This figure represents attribute importance scores estimated using all DCE questions. The vertical bars surrounding each mean preference weight denote the 95% confidence interval (CI) about the point estimate. If the CIs do not overlap for pairs of levels in a particular attribute, the mean estimates are statistically significantly different from each other at the 5% level of significance.

The most important attribute was with whom the participant reviewed the resource (43.9, 95%CI 40.5–47.3), followed by modality (23.4, 95%CI 20.1–26.8) and content (22.6, 95%CI 20.1–25.1) (Fig. 3). Frequency was less important (9.8, 95%CI 7.3–12.3), with the least important attribute being decision-making principles (0.2, 95%CI −2.5 to 2.9).

### Results of the Sensitivity Analyses

Participant characteristics and mammography attitudes were overall similar among all participants who completed the survey (*n* = 819) compared to analysis sample (*n* = 673) (Supplementary Material 3 pages 5–6). For our second sensitivity analysis, preference weight estimates for each RUM as well as importance scores using RPL are reported in Supplementary Material 3, pages 15–21. The results are qualitatively similar to those found using conditional logit. For our final sensitivity analysis, we found evidence that the particiants answered the partial profile differently than the full profile; in the full profile model, the average participant was indifferent to reviewing the resource with their healthcare provider vs. on their own or being delivered as a paper brochure vs. electronic video/website (Supplementary Material 3 pages 8–14).

## DISCUSSION

In our study of preferences for reducing mammography overscreening in older, socioeconomically diverse women, we found that women have strong preferences for how information is delivered and with whom during shared decision-making conversations but not for calculators/checklists that incorporate age/health/preferences/values.

Despite insufficient evidence balancing the harms and benefits of routine mammography screening in women ≥ 75 years old and recommendations to consider health status, life expectancy, and patient wishes in determining whether to discontinue mammography,^1–4^ overscreening continues. Prior research suggests that nearly one-quarter of women have not considered stopping mammograms and one-third meeting criteria for stopping have no intention of doing so.^39^ Our findings suggest that this may be related to the fact that few older women are made aware that mammography is a choice. Our findings also suggest that simple, fact-based patient activation material is an acceptable approach for prompting discussions about whether to discontinue mammography. While conventional wisdom suggests patients prefer emotionally resonant stories/testimonials, it may be that they facts/data outweigh testimonials when deciding whether to stop a health behavior, and should be further explored in future research (e.g., testominials around both continuing and discontinuing mammography may have been more acceptable).

While our study supports the integral role of messaging from healthcare providers,^14,40^ many of whom face time constraints and challenges in adhering to mammography guidelines,^13^ our DCE suggested that older women were also amenable to reviewing the material on their own, either via paper or electronically. This approach may help support more efficient shared decision-making discussions during visits. Research on reducing overscreening has focused on using decision aids,^12^ which may effectively improve knowledge and intention^16^ but face numerous implementation barriers.^41^ In our nationally representative diverse population, use of age/health calculators or checklists of patient wishes/values did not appear to be important in prompting discussions around whether to stop mammograms with their providers. It may be that patients prefer simpler shared-decision making approaches, and future research is needed to identify the efficacy of patient activation/educational approaches and possible unintended consequences. Some women may prefer longer deliberations and decision aids; approaches for elucidating differences in decision-making styles/informational ends should be explored.

Recently, screening guidelines expanded to include women 40–49 years.^42^ Healthcare systems with limited resources will need to weigh (1) reducing overscreening in older patients, particularly those in poor health, and scheduling timely screening and urgent diagnostic tests in younger women against (2) the fact that treatment has become less toxic and more effective among older women,^43,44^ many of whom have a life expectancy ≥ 10 years.^45^ Pairing patient activation with automated approaches for risk-stratifying patients within the EHR may ensure that older patients who might benefit from mammography continue screening.

Our study also advances the field of de-implementation science. According to a recent systematic review, DCEs are increasingly used in implementation science as a method for engaging stakeholders.^21^ Few examples exist of leveraging DCEs as a methodological approach for de-implementation, which is often conceptualized as a complex and multi-faceted process, mostly operating at the organizational or system level.^46^ Emerging data suggests that multicomponent, intrapersonal interventions that address both patient and clinician perspectives have the greatest potential for reducing low-value care.^46,47^ Our study suggests that DCEs can be used to efficiently inform the design and refinement of de-implementation strategies. In fact, we were able to save time by forgoing the creation of a patient story/testimonial (in lieu of just facts), community outreach approach, and risk-based tools, focusing instead on a mailed paper brochure delivered annually. We also found several unique considerations in designing DCEs for de-implementation, including the need to leverage user-centered design principles to refine question stems and display more tangible products within surveys; avoid assumptions around patient awareness of or agreement with guidelines; and simplify and provide more DCE guidance (e.g., we displayed a video describing the DCE and started with partial profiles before delivering the full ones to facilitate understandability). We also included several attention checks, and suggest that deploying full profiles may be particularly difficult for individuals, particularly older populations, to understand as it relates to de-implementation.

Several limitations existed. We used a small existing panel of women ≥ 70 years and results may not be generalizable (e.g., to the U.S. population or those with low digital literacy). Excluding participants without a personal history of breast cancer may have biased our standard errors, though sensitivity analyses revealed similar demographics/attitudes with incusion. It is unclear whether results were impacted by life expectancy, cognitive impairment, or other high-risk features (e.g., BRCA mutation). While our study passed several attention and internal validity checks, we found higher rates of straight-lining (answering all A or all B options), fewer significant results, and larger confidence intervals (i.e., for the importance scores) in the full DCE questions. It may be that respondents were less engaged with the full profiles or that there were only three full profile questions (vs. 12 partial questions), which makes it is naturally more likely that a respondent’s most preferred option will appear in the same location. Nonetheless, nearly all participants passed attention and internal validity checks and most reported general ease and understanding. As such, we included all participants in the final analyses which is considered best practice, avoids selection bias and limits impact on external validity.^48,49^ While the full profile is more statistically efficient and provides more precise parameter estimates for a given number of choice tasks, they are more cognitively demanding; meanwhile partial profiles are simpler but can bias respondent choices by excluding attributes.^50^ By including both full profile and partial profile questions in our survey, we were able to empirically test for differences in our results based on question format with our analyses demonstrating broadly similar results across question formats. Despite oversampling attempts by race/ethnicity, most participants were non-Hispanic White albeit representative of US women ≥ 70 years old and with strong representation from lower socioeconomic levels. Finally, decision-making heuristics were suboptimally considered, many attributes/levels were missing (i.e., preferences for amount of details received) and participants were not shown the content of the patient story/testimonial or pros/cons themselves nor did we explicitly reference/show pictures of decision aids, potentially limiting their ability to distinguish levels (e.g., calculator vs. checklist). Nonetheless, we plan to iteratively design the content and refine decision aids with patients following the DCE.

Overall, our study is one of the first to deploy DCEs to elicit patient preferences for de-implementation strategies. Future research will examine differences in preferences by subgroups and test the impact of our patient activation material, along with provider- and system-level strategies on mammography overscreening.

## Supplementary Information

Below is the link to the electronic supplementary material.

ESM 1 (454 KB PDF)

ESM 2 (25.8 KB DOCX)

ESM 3 (375 KB DOCX)

## Data Availability

Dallas Wood had full access to the data and takes responsibility for the integrity of the data and the accuracy of the data analysis. The datasets used and/or analyzed in this study are available in Open Science, https://www.cos.io upon request.
