# Supplementary material for: Use of a Discrete Choice Experiment to Inform De-implementation of Mammography Overscreening: A US-Based National Survey
Source: J Gen Intern Med. 2026 Jan 27;41(9):2507–17. doi: 10.1007/s11606-025-10158-9 (PMC13305097; doi:10.1007/s11606-025-10158-9)
Supplement: Supplementary file 1 — (454 KB PDF) [file 11606_2025_10158_MOESM1_ESM.pdf]

DE-IMPLEMENTATION OF MAMMOGRAPHY 2024 COLUMBIA UNIVERSITY

PROJECT METHODS AND TRANSPARENCY REPORT

August 16, 2024

Client Contact: Nathalie Moise and Jennifer Mizhquiri Barbecho
NORC Account Manager:
Amy Shin | Shin-Amy@norc.org
NORC Project Manager:
Marissa Slowey | Slowey-Marissa@norc.org

Research You Can Trust™

STUDY INTRODUCTION

NORC conducted the De-implementation of Mammography study on behalf of Columbia University using NORC's AmeriSpeak® Panel for the sample source. This study was funded by the National Cancer Institute. The purpose of the study is to explore strategies to inform and educate older women about the de-implementation of mammography in their healthcare.

The survey was offered in English-only and was self-administered by the respondent online via the Web.

AAPOR Transparency Initiative

This *AmeriSpeak Project Methods and Transparency Report* provides complete information on how the survey was executed, including any information disclosure to meet the requirements of the AAPOR Transparency Initiative. NORC at the University of Chicago is a Charter Member of the AAPOR Transparency Initiative, which fosters open science of survey research by acknowledging those organizations that pledge to practice transparency in their reporting of survey-based research findings. More on the Transparency Initiative can be found here: https://aapor.org/standards-and-ethics/transparency-initiative/

SURVEY OVERVIEW

Study Target Population: Women ages 70+
Sample Units: 2575
Completed Units: 819
Expected Eligibility Rate: 100%
Observed Eligibility Rate: 100%
Margin of Error: ±5.17 percentage points (pp)
Design Effect: 2.28
Survey Field Period: May 9, 2024 – July 22, 2024
Median Duration (minutes): 14

Definitions of the above categories:

Study Target Population: The total set of individuals of interest to which the researcher intends to generalize their conclusions.

Sample Units: The number of panel members selected into the study sample.

Completed Units: The number of sample units that completed the interview based on the study-specific definition of what constitutes a complete interview. This number excludes any cases where an interviewer finished a survey, but the case was removed due to data quality concerns (the process for such removal is detailed later in this report).

Expected Eligibility Rate: The percentage of the sampling population who are expected to meet study eligibility criteria.

Observed Eligibility Rate: The percentage of the sample members who were eligible for the study among those who answered the screening questions.

Design Effect: The design effect is the variance under the complex design divided by the variance under an SRS (simple random sampling) design of the same sample size. The reported design effect is an approximation based on the coefficient of variation of the final survey weights.

Margin of Error: Margin of error is defined as half the width of the 95% confidence interval for a proportion estimate of 50% adjusted for design effect. It is therefore the largest margin of error possible for all estimated percentages based on the study sample.

Survey Field Length: the period from the earliest to the latest contact dates of cases sampled for the survey.

Duration: Length of time for completed interviews. Interview length is calculated differently depending upon whether the interview was conducted over the phone or via the web. For telephone mode, it is the time from when the respondent picks up the telephone until they hang up the telephone. For web interviews, it is the time from when they first connect to the web system to the time they log off the system or become inactive. In the case of multiple contacts, this number represents the sum of those contacts.

STUDY-SPECIFIC DETAILS

Sampling

A sample of women ages 70 and older was selected from NORC's AmeriSpeak Panel for this study. Additionally, an oversample of non-white women ages 70+ was selected for the survey.

The sample for a specific study is selected from the AmeriSpeak Panel using sampling strata based on age, race/Hispanic ethnicity, education, and gender (18 sampling strata in total). Sample selection takes into account the expected differential survey completion rates across the sampling strata. The size of the selected sample per stratum is determined such that the distribution of the complete surveys across the strata matches that of the target population as represented by census data. When panelists are selected for an AmeriSpeak survey, the selection process, within each sampling strata, favors those who were not

selected in the most recent previous AmeriSpeak survey. This selection process is designed to minimize the number of surveys any one panelist is exposed to and maximize the rotation of all panelists across AmeriSpeak surveys.

For more detailed information on the AmeriSpeak panel recruitment and management methodology, please see the Appendix ("Technical Overview of the AmeriSpeak® Panel NORC'S Probability-Based Household Panel") attached to this AmeriSpeak Project Report.

The race/ethnicity targets in this project have the following outcome measures:

Non-Hispanic White

Completed Sample Units (n): 607
Margin of Error: ±6.05 percentage points (pp)
Design Effect: 2.31

Non-Hispanic Black

Completed Sample Units (n): 122
Margin of Error: ±12.89 percentage points (pp)
Design Effect: 2.11

Hispanic

Completed Sample Units (n): 52
Margin of Error: ±19.63 percentage points (pp)
Design Effect: 2.09

All Other

Completed Sample Units (n): 39
Margin of Error: ±20.26 percentage points (pp)
Design Effect: 1.62

Field

A small sample of English-speaking AmeriSpeak web-mode panelists were invited on May 9, 2024 for a soft-launch. In total, NORC collected 59 soft-launch interviews (52 completes, 7 in-progress cases). The initial data from the soft-launch was reviewed by NORC and delivered to Columbia University.

The addition of an attention check and detailed instructions before each of the two discrete choice experiments were made before fielding the remainder of the sample to collect the surveys interviews used for the final data.

The remainder of sampled AmeriSpeak panelists were invited to the survey on May 24, 2024. Data collection ended on July 22, 2024.

In total, NORC collected 819 final interviews. This does not include interviews that may have been removed for data quality purposes (see below).

This final collection of survey completers includes specific oversamples of non-white women ages 70+ (213 total completes) to ensure adequate sample size of those groups for analysis. These oversampled groups are weighted down to match their respective proportion in the population in the weighting process (see description of that process later in this report).

Panel & Survey Sample Performance

To meet requirements in the AAPOR Transparency Initiative, we offer performance outcome measures of both the AmeriSpeak Panel and the sample selected from the panel. The AmeriSpeak Panel is a household panel, so recruitment and retention rates are household rates. The survey sample is an individual-level sample pulled from the AmeriSpeak panel, so those are individual-level rates.

Panel Outcome Measures	
Weighted Household Panel Recruitment Rate (WPRecr)	Weighted Household Panel Retention Rate (WPRet)
15.9%	69.9%

Weighted Household Recruitment Rate (WPRecr): The weighted AAPOR RR3[1] at the household level for AmeriSpeak panel recruitment. A recruited household is a household where at least one adult successfully completed the recruitment survey and joined the panel.

Weighted Household Retention Rate (WPRet): The weighted percent of recruited households that remain on the panel and are available for sampling for this survey. Unavailable panelists are those who have temporarily or permanently asked to be removed from the panel or from receiving surveys.

Survey Sample Outcome Measures	
Survey Completion Rate (SurC)	Weighted Cumulative Response Rate (WCR)
31.8%	3.5%

Survey Completion Rate (SurC): The percent of sample members who completed the survey interview. 2,575 panelists were invited to the survey, and 819 completed the survey. As noted earlier, survey completes exclude any cases removed due to data quality concerns.

Weighted Cumulative Response Rate (WCR): The overall survey response rate that accounts for survey response in all phases, including panel recruitment, panel retention, and survey completion. This overall rate is weighted to account for the sample design and differential inclusion probabilities of sample members in all sampling stages. *(WCR=SurC x WPRet x WPRecr)*

Gaining Cooperation of AmeriSpeak Panelists for the Study

If invited, AmeriSpeak panelists can take the survey online through the password-protected AmeriSpeak Mobile App, the password-protected AmeriSpeak Web portal, or by following a link in the e-mail invitation sent to them.

To encourage study cooperation, NORC sent the initial invitation and email reminders to sampled web-mode panelists on the following dates:

- Thursday, May 9, 2024

[1] AAPOR RR3 and other response rate calculations can be found here: https://www-archive.aapor.org/Education-Resources/For-Researchers/Poll-Survey-FAQ/Response-Rates-An-Overview.aspx.

- Wednesday, May 24, 2024
- Sunday, June 2, 2024
- Friday, July 12, 2024
- Friday, July 17, 2024

Panelists were offered the cash equivalent of $4 for completing this survey.

Data Processing & Data Quality Review

NORC prepared a fully labeled data file of respondent survey data and demographic data for Columbia University.

NORC applied cleaning rules to the survey data for quality control. In total, 1 case was removed from the final set of completed interviews based on three cleaning rules. Descriptions of the cleaning criteria and the counts from each are below (counts are overlapping).

- <u>Removing Speeders</u> (i.e., those that completed the survey in less than one-third the median duration)
  - 1 removed for speeding
- <u>Removing Respondents with High Refusal Rates</u> (i.e., those that skip or refused more than 50% of the eligible questions)
  - 0 removed for high refusal rates

AmeriSpeak is a probability-based panel, where respondents must be chosen by us to join, where access to surveys is controlled by the panelist secure log-in information to a web portal or app. E-mails, text invitations, or interview-operated telephone calls go directly to the address/number of the recruited panelist. When being called by phone, the panelist is requested by name. The way AmeriSpeak surveys are programmed and panelists are invited, panelists cannot take the survey more than once, and each panelist is always identifiable based on a unique ID. For these reasons, AmeriSpeak does not suffer the problem of "bots," fabricated profiles, non-invited respondents, or individuals or members of the household repeatedly and illegitimately taking the same survey.

Statistical Weighting

The final weight variable that is delivered with the data is a product of three weights:

AmeriSpeak Panel Weights: Weights developed for all panel members to account for their probability of selection into the sample of panel recruits, panel recruitment nonresponse adjustments, and poststratification adjustments of the recruited panel to match population benchmarks.

Study Specific Base Weights: Sampling weights developed for a study sample selected from the panel to account for their selection probabilities under the sample design. The base weights are a product of the AmeriSpeak Panel Weights and the inverse of selection probabilities associated with sample selection from the panel.

Study Specific Final Weights: These are final weights developed for all completed cases of a specific study. The final weights are adjustments of the base weights to address survey nonresponse through a weighting class method. Raking adjustments are then applied to the non-response adjusted weights to align the survey sample to specific population benchmarks. The final weights may be trimmed to reduce the influence of extreme weights on survey estimates.

The following information goes deeper into the specifics of each of the weights. You can also find an even deeper discussion of the development of weights in the AmeriSpeak Panel Technical overview report in the Appendix of the Project Methods and Transparency Report.

***AmeriSpeak Panel Weights*:** Since the sampling frame for this study is the AmeriSpeak Panel, which itself is a sample, the starting point of the weighting process for the study is the AmeriSpeak panel weight[2]. To develop the panel weight, NORC first computed the panel base weight as the inverse of the probability of selection from the NORC National Frame (the sampling frame that is used to sample housing units for AmeriSpeak) or other address-based sample frames (supplemental panel samples were selected from frames developed from the USPS Delivery Sequence Files). The sample design and recruitment protocol for the AmeriSpeak Panel involve unequal sampling rates across the sampling strata and additional subsampling of initial nonresponding housing units for in-person nonresponse follow-up (NRFU). The panel base weights reflect all the variations in panel sample selection probabilities. The panel base weights are then adjusted to account for unknown eligibility and nonresponse among eligible housing units. These adjustments were conducted using weighting classes defined by some household characteristics provided by commercial data vendors, including partisan score, political party identification, the presence of young adult(s), and minority status. To produce the final household panel weights, the household-level nonresponse adjusted weights are post-stratified to match the number of households per census division obtained from the most recent Current Population Survey (CPS). Final household weights are assigned to each eligible adult in the recruited household. These person-level weights are then adjusted to compensate for nonresponding adults within a recruited household. Finally, the nonresponse adjusted person-level panel weights are raked to population totals associated with the following variables:

Variables & the Variable Categories for Panel Recruitment Non-Response Raking

Age: 18-24, 25-29, 20-39, 40-49, 50-59, 60-64, and 65+
Gender: Male and Female
Census Division: New England, Middle Atlantic, East North Central, West North Central, South Atlantic, East South Central, West South Central, Mountain, and Pacific
Race: White, Black, AAPI, Other
Ethnicity: Hispanic, Not Hispanic
Education: Less than High School, High School/GED, Some College, and BA and Above
Housing Tenure: Homeowner and Other
Household phone status: Cell Phone-only, Dual User, and Landline-only/Phoneless
Age x Gender: 18-34 Male, 18-34 Female, 35-49 Male, 35-49 Female, 50-64 Male, 50-64 Female, 65+ Male, and 65+ Female
Age x Race/Ethnicity: 18-34 Non-Hispanic White, 18-34 All Other, 35-49 Non-Hispanic White, 35-49 All Other, 50-64 Non-Hispanic White, 50-64 All Other, 65+ Non-Hispanic White, and 65+ All Other
California Adjustment: Californian, non-Californian

The external population totals are obtained from the Current Population Survey, except for Household Phone Status, which is determined by the National Center for Health Statistics (NCHS) bi-annual survey on wireless substitutions.[3] The weights adjusted to the external population totals are the *final panel weights*.

Study Specific Base Weights: These are developed to adjust for unequal selection probabilities from the AmeriSpeak panel, differential nonresponse across subpopulations, and frame coverage limitations. All these weighting adjustments are applied to the final panel weights described above.

The sample for this study is selected from the AmeriSpeak Panel using sampling strata (see the description of the sampling strata for this study earlier in this report). Sample selection takes into

---

[2] The AmeriSpeak panel weight existed prior to this study; the weighting procedures are described here for clarity and completeness.
[3] Blumberg SJ, Luke JV. Wireless substitution: Early Release of Estimates from the National Health Interview Survey, January-June 2022. National Center for Health Statistics. December 2022. Available from: https://www.cdc.gov/nchs/nhis.htm

account the expected differential survey completion rates across these strata based on average completion rates in previous surveys. This sample selection based on expected nonresponse ensures a more representative final sample of completed interviews. However, the net result of the sampling design is an unequal selection probability that varies depending on the strata a respondent represents. *Study-specific base weights* are computed as the product of the final panel weights and the inverse of the probabilities of selection under the study sample design.

Finally, ***Study Specific Final Weights*** are created by first adjusting the base weights for survey nonresponse through a weighting class method, where the weighting classes are defined by age, race/ethnicity, gender, and education. After that, a raking ratio adjustment is applied to the nonresponse adjusted base weights to align the sample with known population benchmarks made up of the topline socio-demographic characteristics of the following:

Variables & the Variable Categories for Study-Specific Survey Non-Response Raking

Race/Ethnicity x Age: Non-Hispanic White and Age 70-74, Non-Hispanic White and Age 75-79, Non-Hispanic White and Age 80+, Non-Hispanic Black and Age 70-74, Non-Hispanic Black and Age 75-79, Non-Hispanic Black and Age 80+, Hispanic and Age 70-74, Hispanic and Age 75-79, Hispanic and Age 80+, Non-Hispanic Other and Age 70-74, Non-Hispanic Other and Age 75-79, Non-Hispanic Other and Age 80+

Race/Ethnicity x Education: Non-Hispanic White and Up to some college, Non-Hispanic White and BA and Above, Non-Hispanic Black and Up to some college, Non-Hispanic Black and BA and Above, Hispanic and Up to some college, Hispanic and BA and Above, Non-Hispanic Other and Up to some college, Non-Hispanic Other and BA and Above

Race/Ethnicity x Region: Non-Hispanic White/All Other and Northeast, Non-Hispanic White/All Other and Midwest, Non-Hispanic White/All Other and South, Non-Hispanic White/All Other and West, Non-Hispanic Black and Northeast, Non-Hispanic Black and Midwest, Non-Hispanic Black and South, Non-Hispanic Black and West, Hispanic and Northeast, Hispanic and Midwest, Hispanic and South, Hispanic and West

These sociodemographic characteristics are weighted to benchmarks from the Current Population Survey.[4].

Raking and re-raking are done during the weighting process so that the weighted demographic distribution of the survey completes resembles the demographic distribution in the target population. The assumption is that the key survey items are related to the demographics. Therefore, by aligning the survey respondent demographics with the target population, the key survey items should also be in closer alignment with the target population.

Survey weights are developed to reduce estimation bias that could arise from unequal selection probabilities, nonresponse, and frame coverage errors. However, excessive weight variation could increase the total sampling error by inflating the variance of the estimates. For that reason, at the final stage of the weighting process, extreme final weights may be trimmed so that extreme weights do not overly influence the survey estimates. Again, a more detailed discussion of our approach to trimming can be found in the Appendix of this report. Weights after trimming are re-raked to the same population totals to produce the ***final study weights***.

[4] The Current Population Survey used is either the February CPS or the March Supplement, usually based on which is most recent.

Additional Oversample Weights

This survey includes oversamples of non-white women ages 70+ which were weighted down to its proportions in the overall population in the final main study weights. Some survey packages are not able to recognize weight variations and do not leverage the full potential of an oversample when testing for statistical significance. The basic SPSS package (without the additional Complex Samples Module) has this limitation, while SAS, Stata, and most R packages do not. Since we are delivering this data in an SPSS format, we have included a second weight variable to address this. The oversample variable in the delivered data has the following variable name: WEIGHT2. The weight values in an oversample weight variable scale up the oversampled group(s) to their actual unweighted sample size. Analyzing the data using this weight variable should only occur when analyzing the oversampled group or any subgroup that is wholly composed of the oversampled group, or when comparing the oversampled with a group outside of that oversample. It is inappropriate to use the oversample weight variable when analyzing the overall survey sample or any subgroup that overlaps (does not fit completely within or without) an oversampled group. Using this weight variable in this inappropriate way will lead to incorrect results that are skewed toward the results of the oversampled groups. It is important to note that, when analyzing the oversampled group, results will be the same whether one is using the oversample weight variable or the main weight variable. This difference is limited to the margin of error attained in data from the oversampled and non-oversampled groups. Without the use of this weight, the margin of error for the oversampled group would be (typically) much larger than the true value, and the margin of error for the non-oversampled group would be lower. In addition, as the main weight will reduce the effective sample size of the oversampled group, it can be the case that using this weight would lead to significant rounding errors, particularly in oversampled of very small populations (e.g., 5%).

Benchmark Comparisons

The following table shows the weighted and unweighted estimates for key demographics and compares them to population benchmarks.[5]

Demographic Category	Subcategory	Unweighted (%)	Weighted (%)	Benchmark (%)
**Age**	70 - 74	25.3	38.1	37.8
	75 - 79	60.2	27.5	27.0
	80 Plus	14.5	34.4	35.2
**Education Status**	Less than High School	1.1	1.4	11.2
	High School Equivalent	15.9	27.8	34.3
	Some College/Associate Degree	36.6	41.1	25.4
	Bachelor's or Higher	46.4	29.7	29.2
**Race/Ethnicity**	Non-Hispanic White	74.1	74.8	74.6
	Non-Hispanic Black	14.9	10.1	9.9
	Hispanic	6.3	8.5	8.9
	All other	4.6	6.7	6.6

As a part of the AAPOR Transparency Initiative, it is incumbent on us to state that there are no perfect studies, and all research and methods have their limitations. The purpose of this document is to make apparent, for this study, some possible limitations, the steps taken to minimize them, and the potential or

[5] Because we trim the weights to remove extreme weights and hold down weight variation, the final study weights may end up deviating from exact populations benchmarks by small but acceptable amounts. Even without trimming, there can be a limit in the ability to perfectly match benchmarks along all variables and categories included in the raking procedure. Our goal is to rake as close as possible before trimming.

known sources of measurable or estimated error whenever possible. However, there is always going to be some unmeasured and unknowable error with all forms of public opinion research, including ours.

Deliverables

The following files were created for Columbia University as part of the study deliverables:

- Survey interview data file in Stata format
- Survey frequency SPSS output in an Excel format (both weighted and unweighted)
- Codebook in an Excel format
- Final questionnaire – in a complete programming format, in Word document
- Final questionnaire – in a simpler format (standard AmeriSpeak intro and outro language, programming language, Spanish (if relevant), and CATI version or interviewer instruction (if relevant) are removed), in Word document
- Project report documenting study procedures and information on the AmeriSpeak Panel

How to Describe AmeriSpeak and NORC @ the University of Chicago

For purposes of publication, when describing the AmeriSpeak Panel and its methodology, we recommend using the following language:

> Funded and operated by NORC at the University of Chicago, **AmeriSpeak**® is a probability-based panel designed to be representative of the US household population. Randomly selected US households are sampled using area probability and address-based sampling, with a known, non-zero probability of selection from the NORC National Sample Frame. These sampled households are then contacted by US mail, telephone, and field interviewers (face to face). The panel provides sample coverage of approximately 97% of the U.S. household population. Those excluded from the sample include people with P.O. Box only addresses, some addresses not listed in the USPS Delivery Sequence File, and some newly constructed dwellings. While most AmeriSpeak households participate in surveys by web, non-internet households can participate in AmeriSpeak surveys by telephone. Households without conventional internet access but having web access via smartphones are allowed to participate in AmeriSpeak surveys by web. AmeriSpeak panelists participate in NORC studies or studies conducted by NORC on behalf of governmental agencies, academic researchers, and media and commercial organizations.
>
> For more information, email AmeriSpeak-BD@norc.org or visit AmeriSpeak.norc.org.

If editors or reviewers are requesting anything more specific or any other detail, please reach out to us to make certain you are using accurate language.

For a less technical, panel-specific description of **AmeriSpeak**, we recommend:

AmeriSpeak is the first U.S. multi-client household panel to combine the speed and cost-effectiveness of panel surveys with enhanced representativeness of the U.S. population, an industry-leading response rate, and an innovative and thorough Project Methods and Transparency Report. Since its founding by NORC at the University of Chicago in 2015, AmeriSpeak has produced more than 1000 surveys, been cited by dozens of media outlets, and become the primary survey partner of the nation's preeminent news service, The Associated Press. AmeriSpeak is the most scientifically rigorous multi-client panel available in the U.S. market. Amerispeak.norc.org.

NORC at the University of Chicago is best described as follows:

NORC at the University of Chicago conducts research and analysis that decision-makers trust. As a nonpartisan research organization and a pioneer in measuring and understanding the world, NORC has studied almost every aspect of the human experience and every major news event for more than eight decades. Today, NORC partners with government, corporate, and nonprofit clients around the world to provide the objectivity and expertise necessary to inform the critical decisions facing society.
www.norc.org
Please refer to the full name “NORC at the University of Chicago” when first mentioning us. Using simply “NORC,” thereafter, is fine. Our name is now only the acronym and does not need to be spelled out.

APPENDIX

TECHNICAL OVERVIEW OF THE AMERISPEAK® PANEL NORC'S PROBABILITY-BASED HOUSEHOLD PANEL

Updated August 13, 2024

NORC prepared this *Technical Overview of the AmeriSpeak Panel* because of our commitment to transparency in research. AmeriSpeak® is a large probability-based household panel funded and operated by NORC at the University of Chicago.

This document covers the following topics:

- Sample Frames for the AmeriSpeak Panel Recruitment
- Sample Selection for AmeriSpeak Panel Recruitment
- Panel Recruitment Procedures
- Transparency in Response Rate Reporting using AAPOR Standards
- Impact of Non-Response Follow-up on Representation of Hard-to-Reach Groups
- Use of Mixed-Mode Data Collection to Represent the Non-Internet and "Net-Averse" Households
- AmeriSpeak Panel Management and Maintenance
- AmeriSpeak Panel Weighting Procedures
- AmeriSpeak Client Study Weighting Procedures

Background

AmeriSpeak is designed to be representative of the U.S. household population, including all 50 states and the District of Columbia. U.S. households are randomly selected with a known, non-zero probability from the NORC National Frame as well as other address-based sample (ABS) frames and then recruited by mail, telephone, and in-person field interviews. AmeriSpeak panelists participate in NORC studies or studies conducted by NORC on behalf of governmental agencies, academic institutions, non-profit organizations, the media, and commercial organizations.

The construction of AmeriSpeak started in October 2014 with pilot samples. In 2015, about 7,000 households were recruited from a sample of around 60,000 addresses. In the ensuing years, approximately 5,000 households have been recruited each year under different sample designs. The current panel size is 65,884 panel members aged 13 and over residing in over 58,147 households.

In addition to the regular panel for general population studies, AmeriSpeak also contains various sub-panels to support studies of special populations, including Amplify AAPI (Asians and Pacific Islanders), AmeriSpeak Latino (Spanish-language-dominant Hispanics), AmeriSpeak Teen (Teen 13-17 years of age), Foresight 50+ (Adults 50 years of age or older), and AmeriSpeak GenForward (Young adults 18-30 with oversamples of African Americans, Hispanics, and Asians).[6] AmeriSpeak is the probability sample source for TrueNorth®, the NORC calibration solution for combining probability and non-probability samples for estimation that leverages data from AmeriSpeak, the American Community Survey, Current Population Survey, and other data sources for improved cost and statistical efficiency.[7]

Sample Frames for the AmeriSpeak Panel Recruitment

All sample frames used for constructing the AmeriSpeak Panel are probability-based.

---

[6] AmeriSpeak's Panel Book lists the topics for which we have data for our specialty panels. Also, please see our Amplify AAPI and Foresight 50+ websites for detailed information on these two specialty panels.
[7] Please see our TrueNorth website for more information.

Different sample frames have been used to construct the AmeriSpeak Panel. For the 2014-2023 recruitments, the primary sampling frame for AmeriSpeak is the 2010 NORC National Frame, a multistage probability master sample that fully represents the U.S. household population. We provide a brief description of how the National Frame was constructed after the 2010 Census. The secondary sampling frame is the USPS Delivery Sequence File.

The NORC National Frame. The primary sampling units (PSUs) in the first stage sample selection are 1,917 National Frame Areas (NFAs), each of which is an entire metropolitan area (made up of one or more counties), a county, or a group of counties with a minimum population of 10,000. A total of 126 NFAs are selected in the first stage, including 38 certainty NFAs, 60 non-certainty urban NFAs, and 28 non-certainty non-urban NFAs. The largest 38 NFAs, those with a population of at least 1,543,728 (0.5 percent of the 2010 Census U.S. population), were selected into the National Frame with certainty.

Within the 126 selected NFAs, the secondary sampling units (SSUs) are segments defined from Census tracts or block groups, where each segment contains at least 300 housing units according to the 2010 Census. Within the certainty NFAs, a sample of 896 segments was selected using systematic probability proportional to size (PPS) sampling, where the size of a segment is the number of housing units. Implicit stratification was achieved by sorting the segments by location (NFA, state, and county), principal city indicator, and by ethnic and income indicators. From each non-certainty urban and rural NFA, a sample of 8 and 5 segments was selected, respectively, using systematic PPS sampling where the measure of size is the number of housing units per segment. A total of 618 segments are selected from the non-certainty NFAs.[8] Overall, a stratified probability sample of 1,514 segments was selected into the National Frame in the second stage of sampling.

Within the selected segments, all housing units are listed using the U.S. Postal Service Delivery Sequence File (DSF). In the 123 segments where the DSF coverage is deemed inadequate, the DSF address list is enhanced with an in-person field listing to improve coverage. The final National Frame, consisting of all listed households in the sample segments, is estimated to provide over 97 percent coverage of the U.S. household population. It contains almost 3 million households, including over 80,000 rural households that are added through the in-person listing.

The USPS Delivery Sequence File. In addition to NORC's National Frame, the DSF has been used frequently as a supplemental sample frame for AmeriSpeak recruitment sampling. Although nationally representative, the 2010 National Frame does not include households from Alaska, Iowa, North Dakota, and Wyoming. Since 2016, the annual panel recruitment sample has included a small address-based sample for these four states selected from the DSF to ensure AmeriSpeak presence in all U.S. States and Washington, D.C. In 2017, an enhanced DSF frame was also used to develop a new Latino Panel with adequate representation of Spanish-language-dominant Hispanics. Census tracts with a high incidence (at least 30%) of Spanish-dominant Hispanics were targeted for this recruitment. Furthermore, within these Census tracts, households that were flagged as Hispanic based on consumer vendor data (that are typically used for direct-mail marketing) were oversampled. For the 2019 recruitments, the entire sample was selected from the DSF to reduce sample clustering and improve panel representation by state and in areas not covered by the National Frame in general.

National Consumer Address File. In 2021, NORC also recruited into AmeriSpeak a probability sample of persons aged 50 and older using a national consumer address file that was estimated to have 96% coverage of the target population. AmeriSpeak empaneled approximately 6,000 panelists 50 years of age or older through this initiative.

---

[8] A sample of 5 segments was selected from each of the 28 non-urban NFAs. However, 2 sample segments were later subsampled out in Montana due to cost.

Voter Registration Files. Finally, the TargetSmart voter registration database was used as a sampling frame to construct the GenForward Panel in 2016. Although GenForward specifically targeted Hispanic, non-Hispanic Black, and non-Hispanic Asian adults who were 18-30 years of age, it also recruited adults 30+ years of age into the regular AmeriSpeak Panel.

Most active AmeriSpeak households (84.6%) are sourced from the NORC National Frame or standard address-based sampling (USPS DSF), with the remainder sourced from consumer address or voter files, as shown in the table below.

Distribution of Active AmeriSpeak Households by Sample Frame Used for Panel Recruitment (updated July 1, 2024)

Sample Frame	% of Active AmeriSpeak Households
NORC National Frame	61.8%
USPS DSF	22.8%
National Consumer Address File	9.2%
Voter Registration File	6.2%

Sample Selection for the Panel Recruitment

Different sample designs have been used to construct the panel in different recruitment years. For panel sample selection between 2014 and 2018 and in 2020, National Frame segments were stratified into six sampling strata based on the race/ethnicity and age composition of each segment, as below:

- Hispanic, high youth segments.
- Hispanic, not high youth segments.
- Non-Hispanic Black, high youth segments.
- Non-Hispanic Black, not high youth segments.
- Other, high youth segments.
- Other, not high youth segments.

Hispanic segments are those where Hispanics make up at least a third of the population and the Hispanic share in the population is greater than that of non-Hispanic Black. Similarly, non-Hispanic Black segments are those where non-Hispanic Black make up at least a third of the population and the non-Hispanic Black share in the population is greater than that of Hispanics. Finally, High Youth refers to segments in which 18-24-year-old adults are at least 12% of the total adult population. The above stratification is used to oversample housing units in areas with a higher concentration of young adults, Hispanics, and non-Hispanic African Americans. The resulting household sample is referred to as the initial AmeriSpeak sample or sample for initial panel recruitment.

To support the second stage of panel recruitment, initially sampled but nonresponding housing units are subsampled for a nonresponse follow-up (NRFU).[9] At this stage, consumer vendor data are matched to the pending housing units, and housing units that are flagged as having a young adult[10] (18-34 years of age) or

[9] A small fraction of initially nonresponding housing units is not eligible for NRFU, including "hard refusals" and those with an appointment for a call back from NORC.
[10] A young adult flagged household refers to a household where MSG or TargetSmart indicated there was an 18-24-year-old adult in the household. In 2016 and 2017, a slightly different definition was used, and a young adult flagged household was defined as having an 18–34-year-old adult in the household by MSG or 18–30-year-old adult by TargetSmart.

minority (Hispanic,[11] non-Hispanic Black[12]) are oversampled for the NRFU sample. Overall, approximately one in five initially nonresponding housing units are subsampled for NRFU using the same six sampling strata defined above. Due to NRFU, these initially nonresponding housing units have a higher selection probability compared to the housing units that were recruited during the first stage of panel recruitment.

A two-phase state-based ABS sample design was used for the 2019 AmeriSpeak recruitments. NORC's National Frame is designed to represent the U.S. household population nationally. Samples within states are less representative of the state population due to sample clustering with sample NFAs. The primary objective of the 2019 design is to improve state-level representation by selecting the recruitment sample mostly from areas that are outside the National Frame. A stratified systematic sample was selected in the first phase, where each state constituted a sampling stratum, and the sample was allocated to the strata proportional to the square root of the state population. In the second phase, young adults, Hispanics, non-Hispanic Blacks, and conservatives are oversampled based on appended commercial data flags to improve their representation in the panel. Because the 2019 design did not use NRFU face-to-face recruitment, the 2019 design did not involve geographic clustering.

In 2020, AmeriSpeak returned to the "standard" sampling strategy employed in 2014 through 2018, with intentions to conduct a robust NRFU. However, the COVID-19 pandemic prevented NORC from utilizing field interviewers and the NRFU was limited to its usual first stage, a Federal Express mailing to 20% of the total sample. After an analysis of state-level representativity after 2019 recruitment, it was determined that further statewide representativity was needed in four states: WI, MO, WA, and CO. As such, the 2020 sample design also included supplemental samples from these four states selected from the DSF.

It was clear at the start of 2021 that NORC would not immediately be able to conduct in-person interviewing given the ongoing COVID-19 pandemic. However, NORC sought to test new sampling strategies (noted below) early in 2021 in the hopes of documenting their efficacy and continuing and improving on them for the rest of 2021. Additionally, it was hoped that NORC would be able to conduct in-person interviewing in the second half of 2021. Given these considerations, the 2021 recruiting sample was split into five replicates, the first of which was selected from the DSF and released early in the calendar year, while future replicates were sampled from the NORC National Frame and were held until mid-year for recruiting.

At the end of 2020, a major assessment of panel representativeness was conducted to inform the 2021 sampling strategy. This analysis explored panel representativeness by state, but as well explored a full range of demographic variables. Meanwhile, this analysis was conducted both with the full panelist dataset as well as by assessing "effective panelists," a measure of the likely demographic distributions that would occur among complete cases in any typical AmeriSpeak survey. This analysis found that AmeriSpeak could benefit from additional recruits in seven groups: households earning over $200,000, households with children, Hispanics, Hispanics that specifically speak Spanish, African Americans, persons ages 18 to 24, and persons with less than a High School education. As such, the 2021 sample was stratified using NORC Big Data Classifiers (Dutwin et al., 2024),[13] a technique utilizing available consumer and other public Big Data to make predictions on a range of household attributes during survey sampling. Households predicted to have one of these seven attributes were oversampled, while households predicted to only hold persons aged 50 and older, or otherwise not predicted hold someone with one of the seven attributes, were under sampled. This sampling method was tested in the first sampling replicate, and given very positive results, was continued in all other 2021 replicates.

---

[11] A Hispanic flagged household refers to a household where MSG or TargetSmart indicated the presence of a Hispanic adult in the household.

[12] A non-Hispanic Black-flagged household refers to a household where MSG or TargetSmart indicated the presence of a non-Hispanic Black adult in the household.

[13] David Dutwin, Patrick Coyle, Joshua Lerner, Ipek Bilgen, Ned English, Leveraging Predictive Modelling from Multiple Sources of Big Data to Improve Sample Efficiency and Reduce Survey Nonresponse Error, Journal of Survey Statistics and Methodology, Volume 12, Issue 2, April 2024, Pages 435–457, https://doi.org/10.1093/jssam/smad016.

The 2021 Big Data strata are the following, specifically households with a person predicted to be:

- Spanish speakers.
- 50 years of age or under.
- 18-24 years of age.
- With a high school diploma or less.
- With household income over $200K.
- With a child 13-17 in the household.
- With other children in the household.

Additional strata include 1) households not predicted to be in any of the seven categories and 2) households not modeled due to missing vendor data.

In 2021, NORC also recruited into AmeriSpeak a probability sample of persons aged 50 and older using a national consumer address file that was estimated to have 96% coverage of the target population. AmeriSpeak re-empaneled approximately 6,000 panelists 50 years of age or older through this initiative. These Panelist are both a part of the Foresight 50+ and AmeriSpeak Panels.

NORC's strategy of "waiting it out" was effective in 2021, as the sample replicates released mid-year allowed NORC to wait for an effective "COVID window" to conduct in-person interviewing. In short, in-person interviewing commenced after the peak of the Delta variant in 2021 and concluded with the peak of the Omicron variants. NORC was able to conduct a full NRFU in-person effort during this time.

For the 2022 and 2023 recruitments, NORC implemented the same sampling strategy where the sampling strata are defined by Big Data Classifiers predictions.

Panel Recruitment Procedures

AmeriSpeak Panel recruitment is a two-stage process: (i) initial recruitment using USPS mailings, telephone contact, and modest incentives, and (ii) a more elaborate NRFU recruitment using FedEx mailings, enhanced incentives, and in-person visits by NORC field interviewers.

For the initial recruitments, sample households are invited to join AmeriSpeak online by visiting the panel website AmeriSpeak.org or by calling a toll-free telephone line (inbound/outbound supported). Both English and Spanish languages are supported for online and telephone recruitment. The initial recruitment data collection protocol features the following: an over-sized pre-notification postcard, a USPS recruitment package in a 9"x12" envelope (containing a cover letter, a summary of the privacy policy, FAQs, and a study brochure), two follow-up postcards, and contact by NORC's telephone research center for sample units with a matched telephone number.

For the second stage NRFU recruitments, a stratified random sample is selected from the nonrespondents of the initial recruitments. Households sampled for NRFU are sent a new recruitment package by Federal Express with an enhanced incentive offer. Shortly thereafter, NORC field interviewers make personal, face-to-face visits to the pending cases to encourage participation. Once the households are located, the field interviewers administer the recruitment survey in-person using CAPI or else encourage the respondents to complete the recruitment survey online or by telephone.

As shown in the table below, 43.6% of active AmeriSpeak households are sourced from NORC's investment in extensive non-response follow-up of households that initially refused or otherwise did not join AmeriSpeak. In years where NORC employed NRFU (all years except 2019), over half (53.1%) of AmeriSpeak's active households are sourced from NRFU.

Percentage of Active AmeriSpeak Panel Households by Recruitment Protocol: Initial Recruitment Protocol v. Non-Response Follow-up (NRFU)[14]

AmeriSpeak Panel Recruitment Years	Percentage of Active AmeriSpeak Households	
	From Initial Recruitment	From NRFU
NRFU Years 2015-18, 2020-23	46.9%	53.1%
All Years (2015-2023)	56.4%	43.6%

Additional panel statistics with respect to the 2014-2023 recruited households are as follows:

- 94% of the active panelists prefer to do web or online surveys, while 6% prefer to participate in telephone surveys;
- 13% of the recruited households are non-Internet;[15]
- 81% are cell phone only or cell phone mostly;
- 16% are African American and 20% Hispanic; and
- 27% have a household income below $30,000 (compared to CPS benchmark of 14%).[16]

Please see our AmeriSpeak Panel Demographics Report for panel statistics on our active panel members eligible for survey sampling.[17]

Transparency in Response Rate Reporting Using AAPOR Standards

AmeriSpeak is committed to transparency in response rate reporting. A properly calculated all-in, cumulative AAPOR response rate incorporates all sources of nonresponse. In the AmeriSpeak context, the cumulative AAPOR response rate, therefore, takes into account (i) the panel recruitment rate, (ii) the panel retention rate, and (iii) the survey participation rate.[18] AmeriSpeak does not have a source of nonresponse for the "profiling" or "on-boarding" stage since the panel recruitment includes the profiling task (where information is obtained for sample targeting and weighting).

Panel Recruitment. A sample household is considered recruited if at least one adult in the household joins the panel. The weighted household recruitment response rate (AAPOR RR3) is about 6% for initial non-NRFU recruitments and 28% for NRFU recruitments.

We report two recruitment response rates: (i) for all the panel recruitment years (2014-2023) and (ii) for the recruitment years with NRFU (2015-2018 and 2021-2023). Across all recruitment years, the cumulative weighted household response rate is 24.4%; across recruitment years with NRFU, the cumulative weighted

---

[14] Accurate as of July 1, 2024. Please note that 2020 is counted as a NRFU year even though NRFU was limited to the use of Federal Express mailers and enhanced respondent incentives (i.e., not using face-to-face, in-person recruitment). Similarly, 2021 is counted as a NRFU year even though field interviewing was limited due to the on-going Covid pandemic.

[15] The non-internet households (HHs) are those that do not select "High-speed, broadband internet at home (such as cable or DSL)" or "Dial-up internet at home" response options when they are asked "What kind of internet access do you have? Please select all that apply" item in the recruitment survey. The non-internet HHs include those that only use internet on a cell connection or mobile phone.

[16] For transparency purposes, unweighted percentages are presented in this section. Hence, these results do not take into account selection probabilities. The base weighted distributions that take into account selection probabilities can be provided upon request.

[17] Our AmeriSpeak Panel Demographics Report is available here.

[18] A properly calculated cumulative AAPOR response rate for panel-based research takes into account all sources of non-response at each stage of the panel recruitment, management, and survey administration process (see https://www.aapor.org/AAPOR_Main/media/publications/Standard-Definitions20169theditionfinal.pdf, page 48-9). A common misapplication of the term "response rate" in online panel surveys is to represent the survey-specific cooperation rate as the "cumulative survey response rate." *See* "Response Rate Calculation Methodology for Recruitment of a Two-Phase Probability-Based Panel: The Case of AmeriSpeak" authored by Robert Montgomery, J. Michael Dennis, N. Ganesh. The paper is available at https://amerispeak.norc.org/research/.

household response rate is 32.9%.[19] All these response rates are weighted by base weights. For client studies requiring a panel recruitment response rate exceeding 30%, the sampling frame may be restricted to the panelists recruited in the NRFU years. The panel recruitment response rate calculation methodology is compliant with AAPOR Standards and fully documented.[20]

Panel Retention. Panel retention rate is computed as the proportion of the number of recruited and currently active households over the number of recruited households. The cumulative AmeriSpeak panel retention rate is 82.1%.

Survey Participation Rate.[21] The study-specific survey participation rate can vary widely (in the range of 20% to 70%) as a result of the specific parameters of the study protocol, including but not limited to the specific study population, topic salience, study sponsorship, length of field period, length of the survey questionnaire, within-panel sample targeting, use of enhanced gaining cooperation techniques (such as the use of pre-notifications by email and/or USPS postcards), and budget allocated to monetary incentives.

All-In, Cumulative AAPOR Response Rates for Client Surveys. For specific AmeriSpeak client surveys, the all-in, cumulative AAPOR RR3 response rate is typically between 10% to 15% depending on specific study parameters such as target population, survey length, time in the field, salience of subject, and other factors as noted above in documenting study-specific survey completion rates. This all-in, cumulative response rate accounts for the panel recruitment rate, panel retention rate, and survey participation rate.[22]

Impact of Non-Response Follow-up on Representation of Hard-to-Reach Groups

NRFU is instrumental in producing the industry-leading response rate for AmeriSpeak Panel recruitments. Moreover, due to the more intensive effort, NRFU recruitments better represent hard-to-reach groups and therefore make the full panel more representative of the target population. For example, initial recruitments tend to under-represent young adults 18-34 years of age. NRFU recruitments correct for this bias by bringing the age distribution of the panel closer to population benchmarks.

Overall, NRFU recruitments significantly improve the representation of the panel with respect to demographic segments that are under-represented among the respondents to the initial recruitment, including young adults (persons 18 to 34 years of age), African Americans, Hispanics, lower-income households, renters, cellphone-only households, and persons with lower educational attainment (e.g., no college degree). To the extent that these demographic characteristics are correlated with substantive survey variables, NRFU helps to reduce potential nonresponse bias in the sample estimates.

NORC's research indicates that NRFU respondents are indeed somewhat different from initial respondents for many common survey variables. For example, compared to the panelists recruited during the initial stage, NRFU panelists tend to be more conservative politically, more likely to attend church, less interested in current events or topics in the news report, less knowledgeable about science, less likely to be in favor of gun control policies, less likely to read a print newspaper (more likely to read the news online and use

---

[19] The cumulative weighted household response rate is higher than both the weighted initial recruitment response rate and the weighted NRFU response rate because NRFU recruits have much higher base weights. In general, the base weights of NRFU recruits are about five times larger than that of initial recruits.

[20] See http://amerispeak.norc.org/research/Pages/WhitePaper_ResponseRateCalculation_AmeriSpeak_2016.pdf

21 We use these terms interchangeably: "participation rate," "completion rate," and "cooperation rate" as applicable to the final stage of the response rate calculation.

[22] A properly calculated cumulative AAPOR response rate for panel-based research takes into account all sources of non-response at each stage of the panel recruitment, management, and survey administration process (see https://www.aapor.org/AAPOR_Main/media/publications/Standard-Definitions20169theditionfinal.pdf, page 48-9). A common misapplication of the term "response rate" in online panel surveys is to represent the survey-specific cooperation rate as the "cumulative survey response rate." *See* "Response Rate Calculation Methodology for Recruitment of a Two-Phase Probability-Based Panel: The Case of AmeriSpeak" authored by Robert Montgomery, J. Michael Dennis, N. Ganesh. The paper is available at https://amerispeak.norc.org/research/.

social media), more likely to eat at fast-food restaurants, and so on.[23] These observations illustrate that NRFU recruitment is critical for achieving a more balanced panel and for making the substantive estimates in AmeriSpeak studies more accurate. Even though NRFU panelists are more reluctant to complete surveys, the addition of NRFU panelists reduced total absolute bias on average by 5 to 21 percentage points when compared to the initial stage recruits (among examined surveys).[24]

Use of Mixed-Mode Data Collection to Represent the Non-Internet and "Net-Averse" Households

The AmeriSpeak Panel supports mixed-mode data collection to improve the response rate and the representativeness of the complete surveys. During the recruitment survey, AmeriSpeak panelists are offered an opportunity to choose their preferred mode—web or phone—for future participation in AmeriSpeak surveys. A recruited household can consist of both web- and phone-mode panelists. Panelists predominantly prefer web over phone mode. As of February 2024, 96% of the active panelists prefer to do web or online surveys, while 4% prefer to participate in telephone surveys. The telephone mode encompasses panelists without internet access, panelists whose only internet access is via a smartphone, panelists with internet access but are unwilling to share an email address, and panelists who are generally uncomfortable with using the internet.

To the extent that non-internet households or "net averse" persons are different from the rest of the population, mixed-mode surveys have better population coverage and produce more accurate population estimates. NORC's telephone interviewers administer the telephone surveys using a data collection system supporting both the phone and web modes, providing an integrated sample management and data collection platform. For panelists using smartphones for web-mode surveys, the NORC survey system renders an optimized presentation of the survey questions for these mobile users.

AmeriSpeak Panel Management and Maintenance

Panel management and maintenance are crucial for panel health and efficiency. NORC maintains strict panel management rules to limit respondent burden, reduce panel attrition, and minimize the risk of panel fatigue. On average, AmeriSpeak panelists are invited to participate in client studies two to three times a month. AmeriSpeak works with NORC clients to create surveys that provide an appropriate user experience for AmeriSpeak panelists. AmeriSpeak will not field surveys that, in our professional judgment, will result in a poor user experience for our panelists. AmeriSpeak also has a designated website and a telephone number for panelist communications.

Panel maintenance is a dynamic process because the AmeriSpeak Panel is supplemented and refreshed regularly over time to grow the panel, compensate for panel attrition, and improve panel representation for specific subpopulations. For example, the Latino Panel and Teen Panel are created to support studies of Hispanics and teenagers, respectively; the 2019 recruitment is primarily designed to improve sample representation at the state level. As panelists are added or/and removed from the panel, the panel refreshment process takes place to ensure that the refreshed panel fully represents the target population. At each panel refreshment, the base weights are recomputed to reflect the cumulative selection probabilities of households and individuals in all recruitment years and from all sample sources. The base weights are then adjusted for nonresponse during panel recruitments, which is followed by raking adjustments to align the panel weights to known population benchmarks.

AmeriSpeak Panel Weighting Procedures

AmeriSpeak *panel weights*, including both household level and person level weights, are developed to account for the probabilities of selection of the housing units, adjustments for unknown eligibility of the

[23] See "The Undercounted: Measuring the Impact of 'Nonresponse Follow-up' on Research Data and Outcome Measures" authored by Ipek Bilgen, J. Michael Dennis, N. Ganesh. The paper will be soon available at https://amerispeak.norc.org/research/.
[24] *See* "Nonresponse Follow-up Impact on AmeriSpeak Panel Sample Composition and Representativeness" authored by Ipek Bilgen, J. Michael Dennis, N. Ganesh. The paper is available at https://amerispeak.norc.org/research/.

housing units, nonresponse associated with panel recruitments, panel attrition, nonresponse from secondary panel members,[25] and raking ratio adjustments to external population benchmarks. More specifically, the weighting steps for panel weights are as follows, with details provided below:

- Compute household-level base weights.
- Adjustments for unknown eligibility.
- Adjustments for household nonresponse.
- Adjustments to household population benchmarks (this yields the final household-level panel weights).
- Initial person-level weights.
- Adjustments for within household nonresponse.
- Raking ratio adjustments to person-level population benchmarks (this yields the final person-level panel weights).

Household base weights
AmeriSpeak Panel annual recruitments use stratified random samples of housing units selected from the NORC National Frame as well as address-based sample frames developed from the USPS Delivery Sequence File (DSF). Initial household base weights are calculated as the inverse probability of selection of housing units for the combined annual samples. In most recruitment years, nonrespondent households at the end of the initial recruitment phase are subsampled for a nonresponse follow-up (NRFU). These subsampled housing units have their initial base weights adjusted to account for NRFU subsampling. NORC refers to the adjusted household base weights that account for both initial sample selection and NRFU subsampling probabilities as the final base weights associated with the sampled housing units. Household base weights are recomputed at each panel refresh, typically carried out monthly to incorporate newly recruited panelists and other changes to the panel (e.g., dropouts). Final household base weights account for the combined household selection probabilities across all recruitment samples and all recruitment years. We denote the final household base weights as BW_{final}.

Household unknown eligibility adjustments
Sampled addresses that are linked to businesses, vacation homes, vacant properties, homes with no one 18 years of age or over are considered ineligible for recruitment. However, the eligibility status is unknown for a fraction of the sampled housing units. AmeriSpeak uses a weighting class approach to account for housing units with unknown eligibility. To create the adjustment cells under the weighting class approach, we use sample design variables such as sampling strata, recruitment year, and tract-level information of household characteristics obtained from the 5-year ACS and Tract-Level Planning Database. Additional household-level variables are obtained from commercial data vendors.

The following variables are used to define the unknown eligibility adjustment cells:

- TargetSmart Party Affiliation (defined from TargetSmart voter file)
    - Republican
    - Other
- TargetSmart Partisanship Score (defined from TargetSmart voter file)
    - >=80
    - 60-79

[25] Primary panel member refers to the initial recruited adult from the household. Secondary panel member refers to other eligible adults in the same household.

- 40-59
- 20-39
- 0-19
- Missing

- Youth and minority status (defined from appended commercial flags)
  - Young and minority
  - Young
  - Minority
  - Other

For weighting GenForward (a special subpanel of young adults recruited for AmeriSpeak in 2017), the unknown eligibility adjustment cells are defined by the following variables:

- Sampling strata (defined by NORC National Frame segments)
  - Hispanic, high youth segment type
  - Hispanic, not high youth segment type
  - Non-Hispanic Black, high youth segment type
  - Non-Hispanic Black, not high youth segment type
  - Other, high youth segment type
  - Other, not high youth segment type
- Sample source
  - AmeriSpeak
  - GenForward (registered voter file sample age 18 to 34)
- Matching status
  - AmeriSpeak only
  - AmeriSpeak and GenForward
  - GenForward only
- Housing ownership status per census tract
  - Owner occupied housing units greater than 55%
  - Otherwise

Cell collapsing is sometimes utilized to ensure that each cell has at least 20 cases with known eligibility. Within each adjustment cell, base weights for housing units with known eligibility are adjusted upward to represent all housing units. We denote the unknown eligibility adjusted household weights as W_{2j}. Only households with known eligibility have a positive W_{2j}.

Household nonresponse adjustments

Household nonresponse adjustments are needed to compensate for (1) known eligible households that do not complete the recruitment survey and (2) previously recruited households that get reclassified as nonrespondent households due to panel attrition. Panel attrition could result in some household members being withdrawn from the panel. For purposes of weighting, if no other adult in a household remains on the panel after an adult is withdrawn from the panel, the household is considered a nonrespondent household.

AmeriSpeak uses a weighting class approach to adjust the weights from the previous step for household nonresponse. The adjustment cells under the weighting class approach are created via the same method as described in the previous step. The same set of variables listed above are used to define the

nonresponse adjustment cells, although typically more cell collapsing is needed to ensure that each cell has at least 20 respondent households.

Within each nonresponse adjustment cell, weights from the previous step for eligible respondent households are adjusted to represent all eligible households. We denote the household nonresponse adjusted weights as W_{3j}. Only respondent households have a positive W_{3j}.

Raking adjustments to household population benchmarks

The final household weights are developed by applying a raking adjustment to W_{3j}. Separately for each Census Division, W_{3j} is adjusted such that the sum of W_{3j} across all respondent households is equal to the total number of households in the division based on the most recent Current Population Survey (CPS) data. We denote the final household panel weights as W_{4j}.

Person level weights

All adults in the responding households are eligible and invited to join the panel. Therefore, for all eligible adults in the household, as identified by the primary panel member, their initial person-level weight, W_{5ij}, is equal to the final household weight W_{4j}, where *i* denotes eligible adults in respondent household *j*.

Person-level within household nonresponse adjustments

The primary panel member identifies and provides contact information for other eligible adults in the same household, and, subsequently these eligible adults from the same household are contacted and asked to complete the recruitment survey. The within household nonresponse adjustments compensate for person-level nonresponse due to the following:

- Eligible adults in the same household as the primary panel member for whom no contact information is available.
- Eligible adults in the same household as the primary panel member who was contacted for panel recruitment but did not complete the recruitment survey.
- Panel members who were withdrawn from the panel when at least one other adult in the same household continues to be an active panel member.

Within each responding household, weights from the previous step for eligible respondents are multiplied by k_j/k_j^{res}, where k_j is the total number of eligible adults and $k_j^{res}$is the number of respondents in household j. We denote the person-level nonresponse adjusted weights as W_{6ij}. Only person-level respondents have a positive W_{6ij}.

Raking adjustments to derive final person-level panel weights

The final step in deriving person-level panel weights is raking adjustments to person-level population benchmarks obtained from the Current Population Survey (CPS), the American Community Survey (ACS), and the National Health Interview Survey (NHIS). The raking dimensions include the following:

- Age group
  - 18-24
  - 25-29
  - 30-39
  - 40-49
  - 50-59
  - 60-64
  - 65+ years
- Gender
  - Male
  - Female
- Education
  - Less than high school
  - High school graduate
  - Some college or less
  - Bachelor's or above

- Race
  - White
  - Black
  - AAPI
  - Other
- Ethnicity
  - Hispanic
  - Non-Hispanic
- Housing tenure
  - Owner
  - Other
- Household phone status
  - Cell-phone-only
  - Dual user
  - Landline-only/phoneless
- Age by gender
- Age by race/ethnicity
- Census Division
- CA vs Rest of country
  - California
  - Rest of country

Population benchmarks for each dimension are obtained from CPS, although housing tenure and household phone status are obtained from ACS and NHIS, respectively. The raked weights are the final person-level panel weights W_{7ij}.

AmeriSpeak Client Study Weighting Procedures

This section details the standard approach for client surveys using the AmeriSpeak panel. Since some projects can have specific design needs, have unique target populations, or use multiple sample sources beyond just AmeriSpeak, the approach to developing the final client survey sample weights may differ in ways from what is detailed here. If this Technical Overview is in the Appendix of a Project Methods and Transparency Report, the actual and specific process of weighting that client project will be detailed in the main narrative of the report and not necessarily in this Appendix.

AmeriSpeak client study weights are calculated for panelists who complete individual client studies to support approximately unbiased estimation based on samples selected from the AmeriSpeak Panel. Weighting procedures could vary for different studies. In general, client study weights are developed in the following steps.

Base weights

Initial base weights for client study samples are defined as the final person-level panel weights. The initial base weights are adjusted to account for the sample selection probabilities associated with the sampling of AmeriSpeak panelists to the client study sample. For a typical general population study, the sample is selected within 48 strata formed by the cross-classification of the following variables: race/ethnicity (Hispanic, non-Hispanic Black, All Other), age group (18-34, 35-49, 50-64, 65+), education (high school graduate/less than high school, some college/college graduate), and sex. The final base weights are computed as the final person-level panel weights divided by the probability of selection from the panel under the client study sample design. We denote the final base weights for client studies as CW_{1ij}.

Adjustments for screener nonresponse

For client studies that include a screener interview to determine the eligibility of sample members, screener nonresponse adjustments are carried out to compensate for sample members who fail to complete the screener questions. Through screener nonresponse adjustments, the base weights for screener respondents are inflated so they represent both respondents and nonrespondents to the screener interview.

We use a weighting class approach to adjust the base weights for screener respondents to compensate for screener nonrespondents. The specific variables used to define the weighting cells could vary from study to study. In general, the variables include age, gender, education, and race/ethnicity. Within each adjustment cell, base weights for screener respondents are inflated to account for screener nonrespondents. We denote the screener nonresponse adjusted weights as CW_{2ij}.

Adjustments for interview nonresponse

Since not all eligible sampled panelists complete the main survey interview, nonresponse adjustments are needed to compensate for eligible nonrespondents. We again use a weighting class approach where the variables used to define the weighting cells could vary across studies. In general, the weighting cells are defined by age, gender, education, and race/ethnicity. Within each cell, the weight from the previous step is divided by the weighted response rate to derive the interview nonresponse adjusted weights CW_{3ij}.

Raking adjustments

Nonresponse adjusted weights are then calibrated to match population benchmarks through raking ratio adjustments. Raking adjusts the weights such that the marginal weight totals match benchmark totals on a specified set of raking variables. The following person-level characteristics are used in the raking adjustments:

- Age
- Gender
- Census Division
- Race/Ethnicity
- Education
- Age by Gender
- Age by Race/Ethnicity
- Race/Ethnicity by Gender

Population benchmarks for each dimension are obtained from the most recent March CPS supplement. The raked weights are denoted as CW_{4ij}, which are final weights unless weight trimming is applied. For clients who prefer normalized weights, where the sum of the weights is equal to the total number of completed surveys, we derive the normalized weights by dividing CW_{4ij} by its average.

Weight trimming

Survey weights are developed to reduce estimation bias that could arise from unequal selection probabilities, nonresponse, and frame coverage errors. However, excessive weight variation could increase the total sampling error by inflating the variance of the estimates. In general, panel members who live in households that were subsampled for NRFU have larger weights compared to panel members who live in households that were not subsampled for NRFU. The purpose of weight trimming is to reduce the variance while avoiding the introduction of bias in the weighted estimates. After trimming, the weights are re-raked to the same population benchmarks.

For AmeriSpeak studies, weight trimming is embedded in the raking step where the weights are raked such that (1) they agree with external population benchmarks and (2) they have minimum variability. Below is a brief description of the AmeriSpeak raking/trimming process:

Survey weights d_i are adjusted to agree with external population totals, t_x, for a set of variables x. Calibrated weights w_i are derived by minimizing the "distance" between w_i and d_i subject to $\sum w_i x_i = t_x$. Specifically, we minimize,

$$\sum D(w_i, d_i) + \gamma \sum D(w_i, \overline{w}) + \lambda \left(\sum w_i x_i - t_x \right)$$

where λ is the Lagrange multiplier; $(\overline{w})$ is the average weight; γ is a user specified parameter. Setting $\gamma = 0$ yields the standard calibration solution, while setting $\gamma \rightarrow \infty$ yields calibrated weights that completely "ignore" d_i.

Large values of γ yield raked weights are trimmed more aggressively. Typically, AmeriSpeak attempts to choose a value of γ that yields: (a) a study design effect less than 2, (b) MSE for key survey estimates under a weighting approach with trimming ($\gamma > 0$) is less than the MSE for key survey estimates under a weighting approach with no trimming (γ = 0), and (c) value of γ that is as close to 0 as possible (ideally, we choose γ =0.5).

ADDITIONAL RESOURCES

Please see the following resources to learn more about AmeriSpeak:

- AmeriSpeak website
- AmeriSpeak's Panel Book
- AmeriSpeak's Responses to ESOMAR 37

To learn more about AmeriSpeak or to share an RFP, please contact AmeriSpeak at AmeriSpeak-BD@norc.org. Information about AmeriSpeak capabilities and research papers is available online at AmeriSpeak.NORC.org.
