# Supplementary material for: Use of a Discrete Choice Experiment to Inform De-implementation of Mammography Overscreening: A US-Based National Survey
Source: J Gen Intern Med. 2026 Jan 27;41(9):2507–17. doi: 10.1007/s11606-025-10158-9 (PMC13305097; doi:10.1007/s11606-025-10158-9)
Supplement: Supplementary file 2 — (25.8 KB DOCX) [file 11606_2025_10158_MOESM2_ESM.docx]

# **Supplementary Material 2.**

*Procedures for identifying Discrete Choice Experiment (DCE) attributes*

Our DCE design, administration and analyses were informed by the International Society for Pharmacoeconomics and Outcomes Research (ISPOR) checklist for good research practices.^1^ We previously conducted an innovation tournament among radiologists, primary care clinicians and specialists (e.g., oncologists, gynecologists) to select strategies for reducing mammography over-screening.^2^ The highest rated strategies included healthcare system alignment on screening guidelines (e.g., consensus on reminder letter language); integrating electronic health record features aligned with mammography guidelines; clinician education/training; as well as patient-level shared decision-making tools and education to promote patient activation. To better select, refine, and tailor a patient activation strategy, we conducted an iterative process for moving between methodological stages of iterative design (e.g., rapid prototyping of our Rethink Resource) and using DCE as a quantitative method for understanding efficient and important components to implement and refine the resource.^3^ Our research team conducted a series of sessions with patient stakeholders and a creative director to iteratively refine research question/problem and design the DCE, identifying unique considerations for de-implementation and the older participants. As part of this process, we created a (1) carefully constructed description of the guidelines and a question for eliciting guideline acceptability (2) brief video describing a tangible patient activation strategy (the “Rethink Resource”) for educating women and prompting provider/patient discussions and shared decision-making and (3) guide/example for selecting options in a DCE. We arrived at a final DCE question step that asked patients to select the option that would prompt them to *have a discussion with their provider* about whether to stop getting mammograms (vs. which of these options would prompt you to stop getting mammograms).

To arrive at our final set of attributes and levels, we first reviewed our previously conducted qualitative studies on barriers and facilitators to reducing mammography overscreening in older women (among patients and providers) to identify potential attributes and areas of contention.^4,5^ We then conducted 17 one-on-one “strategy refinement” interviews with individuals identified from Columbia University Irving Medical Center affiliated mammography clinic, referrals from primary care champions, and flyers in the community (mean age 80.1 [SD= 3.77]; 100% women, 7 Spanish, 10 English). Finally, we iteratively reviewed versions of the DCE survey with our advisory board of mammography, geriatrics, and implementation science experts (n=11). We focused on areas where there was uncertainty or lack of consensus that would result in actionable refinement of our resource. For example, in our 1:1 strategy interviews, most individuals thought that discussions should involve healthcare providers in clinical settings, but some believed healthcare educators or community/senior centers might be useful contexts. In addition, interviewed patients preferred paper brochures or in-person meetings, but questions emerged about the trustworthiness of TV/news/radio. Many remarked on the need to include “research” backed facts though some participants preferred personalized, patient stories/testimonials of arriving at a decision.

Most importantly, our previously conducted qualitative data distinguished patient-provider communication and educational content from *how* patients arrived at their final decision about whether to continue or discontinue mammography (i.e., based on age and health). Our advisory board/experts noted the importance distinguishing general discussions about patient preferences based on age, health, values and life responsibilities from the use of existing risk calculators/decision aids that estimated life expectancy and mortality to help support the decision. Our overarching goal was to elicit whether a patient would be interested in incorporating decision aids and risk-based/life expectancy calculators, but we employed our user centered design expert and advisory board in carefully distilling down the key concepts of this process. For example, our advisors felt this would be too complex for patients to distinguish calculators for life expectancy vs. cancer risk vs. risk/benefit of continuing/discontinuing mammography) all of which are included in current aids <https://www.decidetogether.info/conversation-aid/en/questions/>. We aimed to distinguish the conversational aid on the decidetogether site (that elicits preferences and values – which our patient stakeholder might be easier to understand as life responsibilities) from the use of risk calculators on the site. In addition, we used the term “checklist” to try to distill down the list of questions, considerations, and pros/cons seen on low literacy eprognosis decision aids. We aimed to simplify as much as possible and were particularly cognizant that we would not be able to describe these terms carefully in a remotely delivered survey.

Finally, we attempted to limit the number of attributes given patient age and capacity. For example, we considered other attributes like cost/insurance, but it was not deemed feasible for us to address or change. We also considered an attribute about who would make the final decision (patient vs. provider vs both) but thought that would be difficult for patients to distinguish from who they reviewed the resource with. The final DCE involved 5 attributes of patient activation de-implementation strategy (“The Rethink Resource”) to prompt discussions with healthcare providers about whether to stop getting mammograms: (a) modality (electronic, paper, in-person, phone), (b) context (reviewed with healthcare provider, group of women [e.g. YWCA,], on own), (c) content (information on pros/cons of mammograms, patient story/testimonial about stopping mammograms), (d) frequency (once, yearly), and (e) decision-making principles (risk calculator, checklist of values and life responsibilities).

1. Bridges JF, Hauber AB, Marshall D, et al. Conjoint analysis applications in health--a checklist: a report of the ISPOR Good Research Practices for Conjoint Analysis Task Force. *Value Health.* 2011;14(4):403-413.

2. Shelton RC, Moise, N, Alexander, S, Burroughs, E, Karr, A, Tehranifar, P. . Application of innovation tournament methods to inform patient, provider and system-level de-implementation strategies for reducing mammography overscreening among older women. Paper presented at: Academy Health Science of Dissemination and Implementation in Healthcare 2024; Washington, DC.

3. Dehmel N, Ran Y, Osborne M, et al. Combining service design and discrete choice experiments for intervention design: An application to weather index insurance. *MethodsX.* 2021;8:101513.

4. Austin J, Tehranifar P, Brotzman L, et al. A Mixed-Methods Study of Multi-Level Factors Influencing Mammography Overuse: Implications for De-implementation. *Implementation Science Communications.* 2021;2(110).

5. Brotzman LE, Shelton RC, Austin JD, et al. "It's something I'll do until I die": A qualitative examination into why older women in the U.S. continue screening mammography. *Cancer Med.* 2022;11(20):3854-3862.
