# Supplementary material for: Use of a Discrete Choice Experiment to Inform De-implementation of Mammography Overscreening: A US-Based National Survey
Source: J Gen Intern Med. 2026 Jan 27;41(9):2507–17. doi: 10.1007/s11606-025-10158-9 (PMC13305097; doi:10.1007/s11606-025-10158-9)
Supplement: Supplementary file 3 — (375 KB DOCX) [file 11606_2025_10158_MOESM3_ESM.docx]

# Supplementary Material 3.

# 1.0 Introduction

The following provides details on the analysis of data collected from the Rethink Resource Discrete Choice Experiment (DCE). The attributes and levels used in the experimental design are reported in Table 1-1. The DCE was composed of 15 choice questions—12 “partial profile” choice questions where respondents considered only 2 attributes at a time and 3 “full profile” choice questions where respondents considered all 5 attributes at the same time. The memo is structured as follows. First, we describe the methods used in our analysis. Second, we present the results of our analysis.

Table 1-1. Attributes and Attribute Levels

| **Attribute** | **Attribute Definition** | **Variable Names and Attribute Levels** |
| --- | --- | --- |
| Review | How to review the information | REVIEW1: The Rethink Resource is reviewed with your healthcare provider.  REVIEW2: The Rethink Resource is reviewed on your own.  REVIEW3: The Rethink Resource is reviewed with a group of women (e.g., in a clinic, faith-based gathering, senior center, YWCA). |
| Modality | Modality of information | MODALITY1: The Rethink Resource is delivered through a paper brochure.  MODALITY2: The Rethink Resource is delivered through an electronic video/website.  MODALITY3: The Rethink Resource is delivered in person or via phone call. |
| Frequency | How often you receive | FREQ1: The Rethink Resource is received every year.  FREQ2: The Rethink Resource is received one time. |
| Content | Education Content | CONTENT1: The Rethink Resource contains information on the pros and cons of stopping mammograms.  CONTENT2: The Rethink Resource contains a story of a woman’s experience with stopping mammograms. |
| Principles | Guiding Principles to Inform the Content | PRINC1: The Rethink Resource includes a calculator based on your age and health to guide your decision on whether or not to stop mammograms.  PRINC2: The Rethink Resource includes a checklist of your preferences and life responsibilities to guide your decision on whether or not to stop mammograms. |

# 2.0 Methods

Data collected from the DCE were used to estimate a random utility model (RUM) that quantified patient preferences for each resource attribute. We estimated a RUM for each question type (i.e., partial profile, full profile, and all questions). Next, the coefficient estimates from each RUM were used to calculate an importance score for each attribute. These analyses were conducted using survey weights prepared by NORC to obtain nationally representative estimates. All analyses were performed in Stata 18.0 (StataCorp, College Station, Texas). In this section, we describe how we checked the quality of the data we collected, how we estimated the random utility model, and how we computed attribute importance scores.

### 2.1 Internal Validity and Data Quality Checks

We conducted the following tests to assess the internal validity and quality of data collected from the DCE.

- **Attention Test Question** – To check whether respondents were paying attention when answering the survey questions, we included a question that simply asked them to select “Blue” from a list of colors. If a respondent did not choose blue, this would suggest they were not reading the survey questions carefully.
- **Straight lining Test** - We identified respondents who always select the same option in each of the DCE questions. The probability the preferred alternative is always Option A or Option B for 15 questions in a row is less than 1%. As a result, if a respondent always chooses Option A or B as the most preferred option, this is evidence that they are not answering the DCE questions carefully. This behavior is referred to as “straight-lining.” We estimated the unweighted percentage of respondents who exhibit straight-lining behavior and compared that estimate with other estimates in the literature. Johnson et al. (2019) found that across 55 health related DCEs that the median proportion of respondents exhibiting straight lining behavior was 2% and the interquartile range was 1% to 8%.
- **Respondent Self-Reported Experience Answering DCE Questions** - After the partial and full profile choice questions, each respondent was asked a series of “debrief” questions designed to collect data on how they felt about the DCE questions. First, the respondent was asked to rate their agreement with the statement “The questions were easy to understand,” on a scale from “strongly agree” to “strongly disagree.” Next, the respondent was asked to rate their agreement with the statement “I found it easy to answer all the questions” on a scale from “strongly agree to strongly disagree.” These two questions were based on identical questions included in Janssen et al. (2018). We calculated the unweighted percentage of respondents that agreed with these statements and compared them to results found in Janssen et al. (2018). Specifically, Janssen et al. (2018) found that 88% of their respondents somewhat or strongly agreed that the DCE questions were easy to understand and 84% somewhat or strongly agreed that the DCE were easy to understand.

### 2.2 Estimating the Random Utility Model

To estimate the RUM, we assumed that each respondent selected the resource option that provides the highest level of utility. We defined the utility that a person *i* receives from hypothetical Rethink Resource option *j* by

u_ij_ = v_ij_ + ε_ij_ (1)

where *v_j_* is the observable component of utility that depends on the option’s attribute levels. The term ε*_j_* is a random error representing the component of utility that is unobservable from the perspective of the analyst but known to the individual.

Under the assumption of utility maximization, person *i* will choose option *j* over option *k* in a given choice task if *u_ij_* ≥ *u_ik_* ∀*k* ≠ *j*. Because total utility is unobserved by the analyst, this choice is random from the perspective of the model, and we can only state the probability that option *j* will be chosen. In general terms, this probability is given by:

Pr(u_ij_ > u_ik_) = Pr(v_ij_ + ε_ij_ > v_ik_ + ε_ik_) = Pr(ε_ik_ – ε_ij_ < v_ij_ – v_ik_) (2)

How this model is estimated depends on the assumptions that are made for the observable component of the utility function and the error term. We describe our estimation approach below.

For the purposes of this study, we estimated the following indirect utility function:

v = β_REVIEW1_ × REVIEW1 + β_REVIEW2_ × REVIEW2 + β_MODALITY1_ × MODALITY1 + β_MODALITY2_ × MODALITY2 + β_FREQ1_ × FREQ1 + β_CONTENT1_ × CONTENT1 + β_PRINC1_ × PRINC1 (3)

All independent variables in this equation were represented by dummy-coded variables for the attribute levels. The coefficients of equation 3 are often called “preference weights.” These coefficients reflect how much satisfaction a respondent receives from attribute level over the excluded attribute level. We also converted all preference weights to Odds Ratios by exponentiation of utilities. ORs corresponded to the odds of an individual preferring a hypothetical Rethink Resource with a given design attribute versus an alternative hypothetical Rethink Resource with a different design attribute. We estimated all preference weights using conditional logit regression. Conditional logit is the basic model for analyzing data generated from DCE and was first estimator shown to be consistent with random utility theory (Hauber, 2016).

As a **sensitivity check**, we also estimated the preference weights discussed above using random-parameters logit (RPL). The RPL was estimated using simulated maximum likelihood estimation with 500 Halton draws and assuming the distribution around each parameter is normal and independent. We implemented this analysis using the *mixlogit* command in Stata 18.0 (Hole, 2007). A key advantage of RPL over conditional logit is that RPL avoids bias created by systematic variations in preferences across respondents. RPL avoids this potential bias by estimating a distribution of preferences around each model parameter that accounts for variations among individual preferences not accounted for by the variables in the model (McFadden & Train, 2000).

### 2.3 Calculating Attribute Importance

We quantified how important each attribute was to the average patient’s resource choice using the overall attribute importance score. This score was calculated by first calculating the difference between the most preferred level and the least preferred level of each attribute. This is obtained from the estimated preference weights. Next, we added these differences and calculated what proportion of the sum was associated with each attribute (Gonzalez, 2019). The larger the importance score, the more important the attribute is to the average patient. The standard errors and the 95% confidence interval (CI) for these importance scores was calculated using the delta method. If the CIs between attributes do not overlap, the importance scores are significantly different from each other at the 5% significance level.

### 2.4 Exploring Differences in Preferences by Question Type

As noted above, the DCE included two types of question: 12 partial profile questions and 3 full profile questions. As part of this analysis, we explored whether the preferences captured using the partial profile questions differ from the preferences captured using the full profile questions. We explored this question using the log likelihood ratio test to determine whether the same preference structure underlies the data collected from each question type. The null hypothesis is that preference weights are equivalent across each question type. If the null hypothesis is rejected, this would suggest that preferences vary depending on how the question was asked. An α level of 0.05 (or 5%) was used to evaluate statistical significance. This log likelihood ratio test was conducted by estimating a conditional logit model for each question type separately.

# 3.0 Results

### 3.1.1 Sample Characteristics

Table 3-1 provides descriptive statistics of our sample.

**Table 3‑1.2**

|  | **Population Characteristics (n=819)** | | | **Sample Characteristics (*n* = 673) excluding those with a history of breast cancer** | | |
| --- | --- | --- | --- | --- | --- | --- |
| **Characteristics** | **Number of Respondents** | **Unweighted Percentage** | **Weighted  Percentage** | **Number of Respondents** | **Unweighted Percentage** | **Weighted  Percentage** |
| **Age** |  |  |  |  |  |  |
| 70-74 years old | 207 | 25.3 | 38.1 | 161 | 23.9 | 34.0 |
| 75-80 years old | 493 | 60.2 | 27.5 | 421 | 62.6 | 31.0 |
| 80 years old or older | 119 | 14.5 | 34.4 | 91 | 13.5 | 35.0 |
| **Race/Ethnicity** |  |  |  |  |  |  |
| White, non-Hispanic | 607 | 74.1 | 74.8 | 493 | 73.3 | 72.0 |
| Black, non-Hispanic | 122 | 14.9 | 10.1 | 100 | 14.9 | 10.5 |
| Other Non-Hispanic (Asian, 2+ Races, etc.) | 38 | 4.6 | 6.7 | 33 | 4.9 | 7.7 |
| Hispanic | 52 | 6.3 | 8.5 | 47 | 7.0 | 9.8 |
| **Education** |  |  |  |  |  |  |
| Less than high school | 9 | 1.1 | 1.4 | 7 | 1.0 | 1.3 |
| High school or equivalent (or GED) | 130 | 15.9 | 27.8 | 103 | 15.3 | 26.0 |
| Some college or Associate’s degree (e.g., AA, AS) | 300 | 36.6 | 41.1 | 253 | 37.6 | 42.0 |
| Bachelor’s degree (e.g., BA, BS) | 174 | 21.3 | 14.3 | 152 | 22.6 | 15.6 |
| Post graduate study or professional degree | 206 | 25.2 | 15.4 | 158 | 23.5 | 15.1 |
| **Marital Status** |  |  |  |  |  |  |
| Married | 381 | 46.5 | 44.8 | 306 | 45.5 | 41.2 |
| Not Married | 438 | 53.5 | 55.2 | 367 | 54.5 | 58.8 |
| **Household Income** |  |  |  |  |  |  |
| Less than $30,000 | 153 | 18.7 | 18.9 | 139 | 20.7 | 22.1 |
| $30,000 to under $60,000 | 298 | 36.4 | 40.2 | 250 | 37.2 | 40.6 |
| $60,000 to under $100,000 | 196 | 23.9 | 24.1 | 154 | 22.9 | 23.0 |
| $100,000 or more | 172 | 21.0 | 16.8 | 130 | 19.3 | 14.3 |
| **Region** |  |  |  |  |  |  |
| Northeast | 114 | 13.9 | 17.8 | 92 | 13.7 | 17.0 |
| Midwest | 199 | 24.3 | 21.8 | 160 | 23.8 | 20.7 |
| South | 272 | 33.2 | 37.2 | 223 | 33.1 | 38.7 |
| West | 234 | 28.6 | 23.2 | 198 | 29.4 | 23.7 |
| **Would you say your health in general is?** |  |  |  |  |  |  |
| Excellent | 50 | 6.1 | 5.5 | 49 | 7.3 | 6.9 |
| Very good | 324 | 39.6 | 37.9 | 274 | 40.7 | 39.3 |
| Good | 326 | 39.8 | 42.5 | 258 | 38.3 | 39.9 |
| Fair | 103 | 12.6 | 12.6 | 78 | 11.6 | 12.1 |
| Poor | 13 | 1.6 | 1.3 | 11 | 1.6 | 1.5 |
| Skipped | 3 | 0.4 | 0.2 | 3 | 0.5 | 0.3 |
| **First degree biological relative diagnosed with breast cancer** |  |  |  |  |  |  |
| Yes | 199 | 24.3 | 28.9 | 151 | 22.4 | 24.0 |
| No | 600 | 73.3 | 67.3 | 509 | 75.6 | 71.8 |
| Not Sure | 18 | 2.2 | 3.6 | 12 | 1.8 | 4.1 |
| Skipped | 2 | 0.2 | 0.1 | 1 | 0.2 | 0.1 |
| **Last mammogram** |  |  |  |  |  |  |
| A year ago or less | 524 | 64.0 | 60.5 | 417 | 62.0 | 56.7 |
| More than 1 year, up to 2 years ago | 110 | 13.4 | 12.3 | 100 | 14.9 | 14.5 |
| More than 2 years, up to 3 years ago | 50 | 6.1 | 6.2 | 47 | 7.0 | 7.8 |
| More than 3 years ago | 117 | 14.3 | 19.2 | 92 | 13.7 | 18.9 |
| I've never had a mammogram | 17 | 2.1 | 1.7 | 16 | 2.4 | 2.1 |
| Skipped | 1 | 0.1 | 0.0 | 1 | 0.2 | 0.1 |
| **Mammogram Planning**  Plan to get a mammogram every 1 -2 years or so  Don’t plan to get mammogram in next few years  I’ve decided to stop altogether  I’m undecided/Other | 622  45  70  82 | 75.9  5.5  8.5  10.0 | 71.7  7.2  10.4  10.7 | 512  40  65  56 | 76.1  5.9  9.7  8.3 | 70.8  8.2  13.0  8.0 |
| **Did a healthcare provider told you that you could choose whether or not to have a mammogram?**  Yes  No  Skipped | 301  516  2 | 36.8  63.0  0.2 | 37.9  61.8  0.3 | 251  420  2 | 37.3  62.4  0.3 | 38.8  60.9  0.4 |
| **Agreement with idea of stopping mammograms based on a woman’s age and health**  Strongly/Somewhat Agree  Neutral  Somewhat/Strongly Disagree | 362  159  298 | 44.2  19.4  36.4 | 45.0  18.2  36.8 | 316  139  218 | 47.0  20.7  32.4 | 49.6  20.9  29.5 |

Source: 2024 De-implementation of Mammography Survey

### 3.2. Internal Validity Tests

Tables 3-2 reports presents the results of the internal validity tests we conducted to assess the quality of data we collected. We found few respondents failed the attention test question. This suggests most respondents were paying attention to the survey.

We also found that the few respondents “straight lined” the partial profile questions in the survey (i.e., always chose Option A or Option B). This suggests that most respondents understood these choice questions and answered them thoughtfully. However, we do see a significant increase in the failure rate when considering the full profile questions. It is important to note that part of this increase is due to that fact there were only three full profile questions, so it is naturally more likely that a respondent’s most preferred option will appear in the same location. Specifically, the probability that more preferred alternative is always Option A or Option B in 3 randomly positioned pairwise comparisons is 12.5%. However, this increase may also suggest that respondents had a harder time understanding and answering these questions.

**Table 3‑2. Percentage of Respondents Failing Internal Validity Tests**

| **Internal Validity Test** | **Expected Failure Rate^a^** | **Observed Failure Rate (Unweighted)** |
| --- | --- | --- |
| Attention Test  Question | N/A | 3  (0.4%) |
| Straight-lining  (Partial profile questions) | 1%-8% | 20  (3.0%) |
| Straight-lining  (Full profile questions) | 1%-8% | 181  (26.9%) |
| Straight-lining  (All choice questions) | 1%-8% | 15  (2.2%) |

^a^ Expected failure rates are the interquartile range of failure rates observed in 55 health related DCE for these internal validity tests (Johnson et al., 2019).
Source: 2024 De-implementation of Mammography Survey, *N* = 673.

Table 3-3 reports the results for questions on respondent experience answering DCE questions. We found for both partial profile and full profile DCE questions, the vast majority of respondents found the DCE questions easy to understand and answer. It is encouraging that respondents found the questions easy to understand and answer. However, this is not what we would expect if the higher failure rate of straight lining for full profile DCE questions was due to their increased difficulty.

**Table 3‑3. Respondent Experience Answering DCE Questions**

| **Statement** | **Respondents Who Strongly Agree or Agree (Unweighted Percentage)** |
| --- | --- |
| **Experience Answering Partial Profile DCE Questions** |  |
| I found it easy to understand the questions above | 526  (78.2%) |
| I found it easy to answer all the questions above | 486  (72.2%) |
| **Experience Answering Full Profile DCE Questions** |  |
| I found it easy to understand the questions above | 533  (79.2%) |
| I found it easy to answer all the questions above | 485  (72.1%) |

Source: 2024 De-implementation of Mammography Survey, *N* = 673.

### 3.3. Preference Weights

Table 3-4 reports preference weights estimated for three RUMS: 1) one estimated using only partial profile DCE questions, 2) one using only full profile DCE questions, and 3) one using all DCE questions. The preference weights from each model are illustrated in Figure 3-1, 3-2 as well as Figure 1 in the manuscript. Across all three models, we find:

- Respondents prefer to review the resource with a health care provider over reviewing the resource with a group of women.
- Respondents prefer to review the resource on their own over reviewing the resource with a group of women.
- Respondents prefer the resource to be delivered as a paper brochure over being delivered in-person or over the phone.
- Respondents prefer the resource to be delivered as an electronic video/website over being delivered in-person or over the phone.
- Respondents prefer to receive the resource every year over a single time.
- Respondents prefer the resource to contain information on the pros and cons of continuing to get mammograms over a story of a woman’s experience.
- Respondents had no preference over whether the resource included a calculator a checklist.

The results of the likelihood ratio test indicated that the null hypothesis of equal preference weights across question type could be rejected at the 1% significance level. This suggested that respondents answered the partial profile questions differently from the full profile questions. In particular, for the full profile model, we find that the average respondent is indifferent between reviewing the resource with their healthcare provider or reviewing the resource on their own. Similarly, for the full profile model, we find the average respondent is indifferent between the resource materials being delivered as a paper brochure or being delivered as an electronic video/website.

Preference weights estimates for each RUM using RPL are presented in Table A-1 and illustrated in Figure A-1, A-2, and A-3. The results are qualitatively similar to those found using conditional logit. However, it is worth noting that results for RUM estimated using only the full profile questions are no longer statistically significant when using the RPL estimator.

Table 3‑4. **Conditional Logit Results (Weighted)**

|  | **Partial Profile DCE Questions Only** | | **Full Profile DCE Questions Only** | | **All DCE Questions** | |
| --- | --- | --- | --- | --- | --- | --- |
|  | **Mean  (Std. Err.)** | **Odds  Ratio** | **Mean  (Std. Err.)** | **Odds  Ratio** | **Mean  (Std. Err.)** | **Odds  Ratio** |
| How to Review the Information | | | | | | |
| Reviewed with your healthcare provider v. reviewed with a group of women (REVIEW1 v. REVIEW3) | 1.76*†  (0.10) | 5.8 | 1.07* (0.11) | 2.9 | 1.52*† (0.08) | 4.6 |
| Reviewed on your own v. reviewed with a group of women (REVIEW2 v. REVIEW3) | 1.46*†  (0.09) | 4.3 | 0.89* (0.12) | 2.4 | 1.22*† (0.07) | 3.4 |
| Modality of Information | | | | | | |
| Delivered through a paper brochure v. delivered in person or via phone call (MODALITY1 v. MODALITY3) | 0.98*†  (0.09) | 2.7 | 0.49* (0.13) | 1.6 | 0.81*† (0.07) | 2.3 |
| Delivered through an electronic video/website v. delivered in person or via phone call (MODALITY2 v. MODALITY3) | 0.73*† (0.08) | 2.1 | 0.35* (0.13) | 1.4 | 0.60*† (0.07) | 1.8 |
| How Often You Receive | | | | | | |
| Received every year v. received one time (FREQ1 v. FREQ2) | 0.36* (0.06) | 1.4 | 0.27* (0.08) | 1.3 | 0.34* (0.05) | 1.4 |
| Education Content | | | | | | |
| Information on the pros and cons of stopping mammograms v. a story of a woman’s experience with stopping mammograms (EDUC1 v. EDUC2) | 0.99* (0.06) | 2.7 | 0.38* (0.08) | 1.5 | 0.79* (0.05) | 2.2 |
| Guiding Principles to Inform the Content | | | | | | |
| Calculator based on your age and health v. checklist of your preferences and life responsibilities (PRINC1 v. PRINC2) | 0.01 (0.06) | 1.0 | -0.03 (0.08) | 1.0 | -0.01 (0.05) | 1.0 |

Source: 2024 De-implementation of Mammography Survey, *N* = 673.

Notes: Standard errors are in parentheses. A star (*) denotes the coefficient is statistically different from zero at the 5% significant level. A cross (†) denotes that two coefficients for the same attribute are statistically different from each other at the 5% significance level.

Figure 3-1. Conditional Logit Preference Weights (Partial Profile DCE Questions Only)


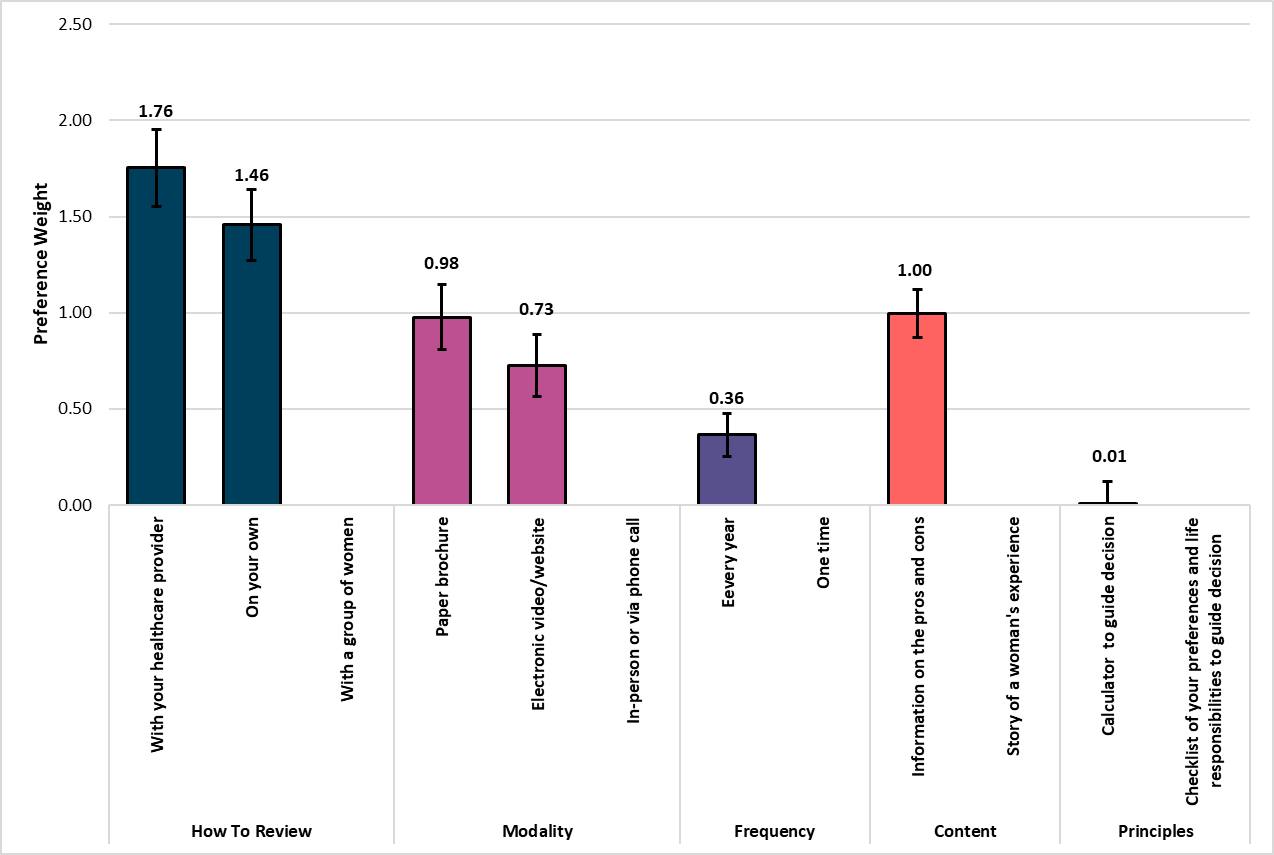


Source: 2024 De-implementation of Mammography Survey, *N* = 673.

Figure 3-2. Conditional Logit Preference Weights (Full Profile DCE Questions Only)


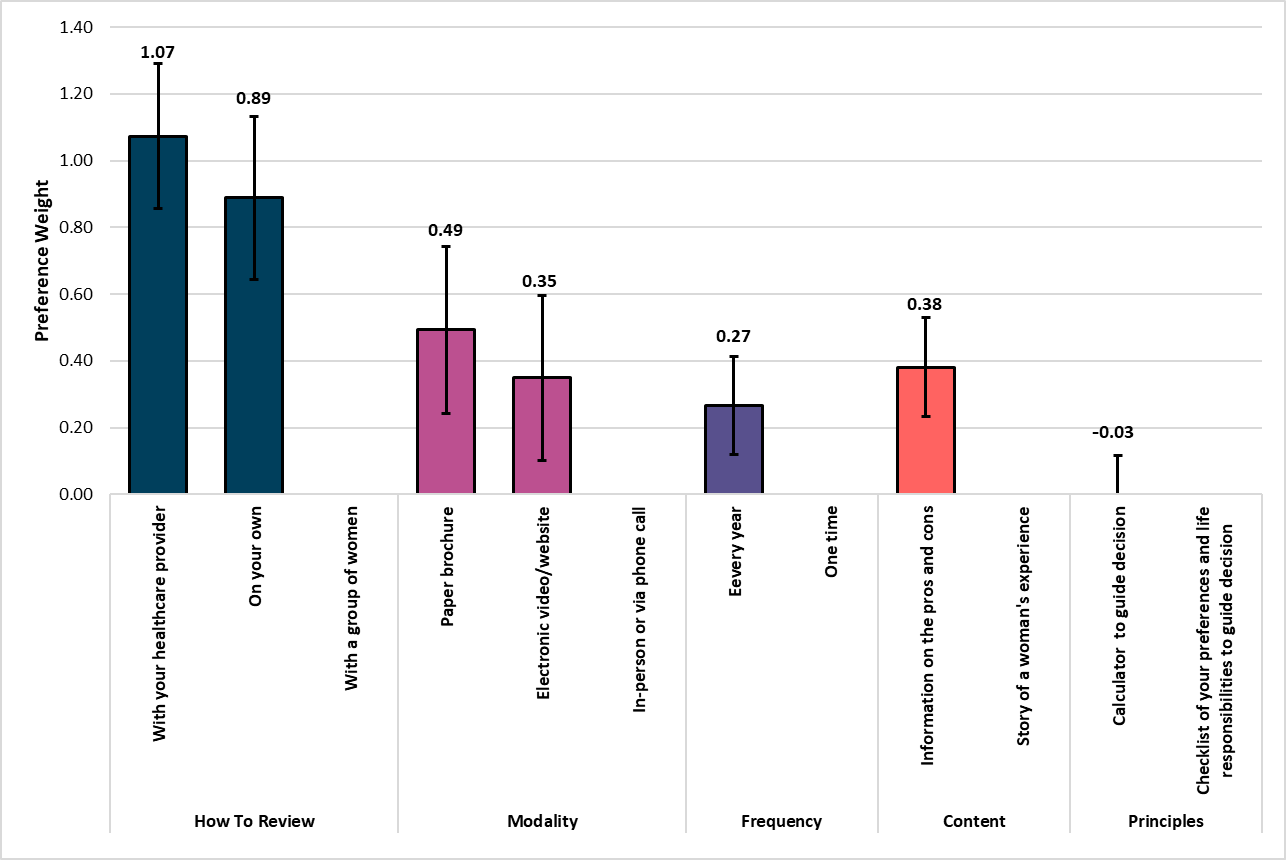


Source: 2024 De-implementation of Mammography Survey, *N* = 673.

Figure 3-3. Conditional Logit Preference Weights (All DCE Questions)

See manuscript for figure.

### 3.4. Attribute Importance Scores

Table 3-5 reports attribute importance scores derived from the preference weights above for each risk range group. These importance scores for all RUMS are illustrated in Figure 3-4. The importance scores the RUM estimated using all DCE questions is illustrated in Figure 2 in the Manuscript. As one can see, the most important attribute was who the respondent reviewed the resource with, and the least important attribute was the guiding principles to inform content. This was true across all 3 models. However, we do see some differences.

For the model using all DCE questions and the model using only partial profile DCE questions, we find the average respondent ranks the resource attributes in the following order:

1. How to review the information
2. Education content = Modality of information
3. Frequency
4. Guiding Principles

For the model using only full profile DCE questions, we find the average responds ranks resource attributes in the following order:

1. How to review the information
2. Education content = Modality of information = Frequency
3. Guiding Principles

The primary difference between these two sets of results is that the importance scores derived from full profile DCE questions have larger standard errors than the importance scores derived from the partial profile DCE questions. This is likely due to the fact that fewer full profile questions were asked and thus the RUM was estimated using fewer observations.

Attribute importance scores estimated using RPL are presented in Table A-2. These importance scores for all RUMS are illustrated in Figure A-4. The importance scores the RUM estimated using all DCE questions is illustrated in Figure A-5. As one can see, these results are similar to those found using conditional logit.

Table 3‑5. **Attribute Importance Score by RUM Model Estimated Using Conditional Logit**

| Attribute | Partial Profile DCE Questions Only | Full Profile DCE Questions Only | All DCE Questions |
| --- | --- | --- | --- |
| How to review the information | 42.8 [39.1 - 46.6] | 47.8 [39.4 - 56.2] | 43.9 [40.5 - 47.3] |
| Modality of Information | 23.8 [20.4 - 27.2] | 22.0 [12.7 - 31.3] | 23.4 [20.1 - 26.8] |
| Frequency | 8.9 [6.3 - 11.5] | 11.9 [5.8 - 17.9] | 9.8 [7.3 - 12.3] |
| Education Content | 24.3 [21.6 - 27] | 17 [10.9 - 23.1] | 22.6 [20.1 - 25.1] |
| Guiding Principles to Inform the Content | 0.2 [-2.6 - 3] | 1.4 [-5.1 - 7.8] | 0.2 [-2.5 - 2.9] |

Source : 2024 De-implementation of Mammography Survey, *N* = 673.

Note: The 95% confidence interval (CI) is included in brackets. When the 95% CI around a mean importance score includes zero, the mean importance is not statistically different from zero. Also, if the 95% CIs around 2 different importance scores do not overlap, then the mean scores are statistically significantly different at the 5% level.

**Figure 3-4. Attribute Importance Scores by RUM Model Estimated Using Conditional Logit**

This figure represents attribute importance scores by RUM model. The vertical bars surrounding each mean preference weight denote the 95% confidence interval (CI) about the point estimate. If the CIs do not overlap for pairs of levels in a particular attribute, the mean estimates are statistically significantly different from each other at the 5% level of significance.


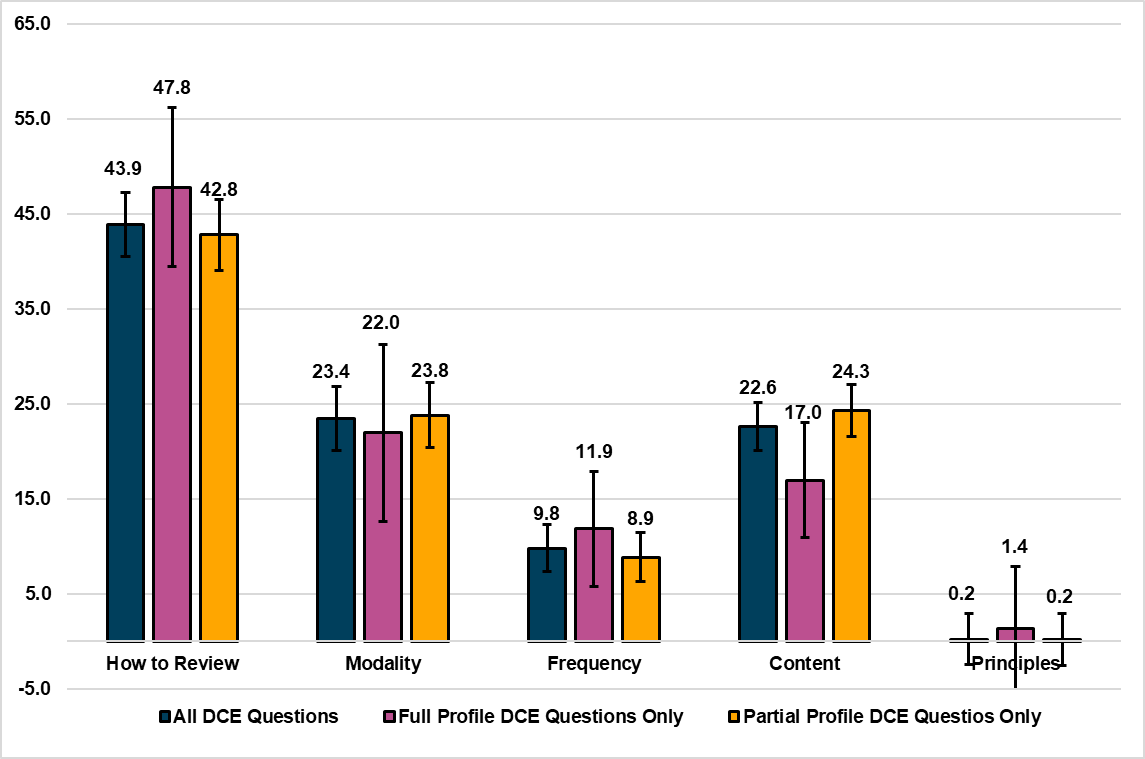


Source: 2024 De-implementation of Mammography Survey, *N* = 673.

Appendix A: Random-Parameters Logit Results

Table A-1. Random-Parameters **Logit Results (Weighted)**

|  | **Partial Profile DCE Questions Only** | | **Full Profile DCE Questions Only** | | **All DCE Questions** | |
| --- | --- | --- | --- | --- | --- | --- |
|  | **Mean  (Std. Err.)** | **Odds  Ratio** | **Mean  (Std. Err.)** | **Odds  Ratio** | **Mean  (Std. Err.)** | **Odds  Ratio** |
| How to Review the Information | | | | | | |
| Reviewed with your healthcare provider v. reviewed with a group of women | 3.12*†  (0.32) | 22.6 | 2.89 (2.51) | 18.0 | 2.77*† (0.22) | 15.9 |
| Reviewed on your own v. reviewed with a group of women | 2.41*†  (0.24) | 11.2 | 2.39 (2.14) | 10.9 | 2.17*† (0.19) | 8.7 |
| Modality of Information | | | | | | |
| Delivered through a paper brochure v. delivered in person or via phone call | 1.67*†  (0.20) | 5.3 | 1.39 (1.15) | 4.0 | 1.43*† (0.16) | 4.2 |
| Delivered through an electronic video/website v. delivered in person or via phone call | 1.24*† (0.18) | 3.4 | 1.09 (0.98) | 3.0 | 1.04*† (0.14) | 2.8 |
| How Often You Receive | | | | | | |
| Received every year v. received one time | 0.68* (0.15) | 2.0 | 0.78 (0.66) | 2.2 | 0.64* (0.13) | 1.9 |
| Education Content | | | | | | |
| Information on the pros and cons of stopping mammograms v. a story of a woman’s experience with stopping mammograms | 1.64* (0.16) | 5.1 | 0.92 (0.85) | 2.5 | 1.29* (0.12) | 3.6 |
| Guiding Principles to Inform the Content | | | | | | |
| Calculator based on your age and health v. checklist of your preferences and life responsibilities | 0.05 (0.11) | 1.1 | -0.06 (0.18) | 0.9 | 0.04 (0.09) | 1.0 |

Source: 2024 De-implementation of Mammography Survey, *N* = 673.

Notes: Standard errors are in parentheses. A star (*) denotes the coefficient is statistically different from zero at the 5% significant level. A cross (†) denotes that two coefficients for the same attribute are statistically different from each other at the 5% significance level.

Figure A-1. Random-Parameters Logit Preference Weights (Partial Profile DCE Questions Only)


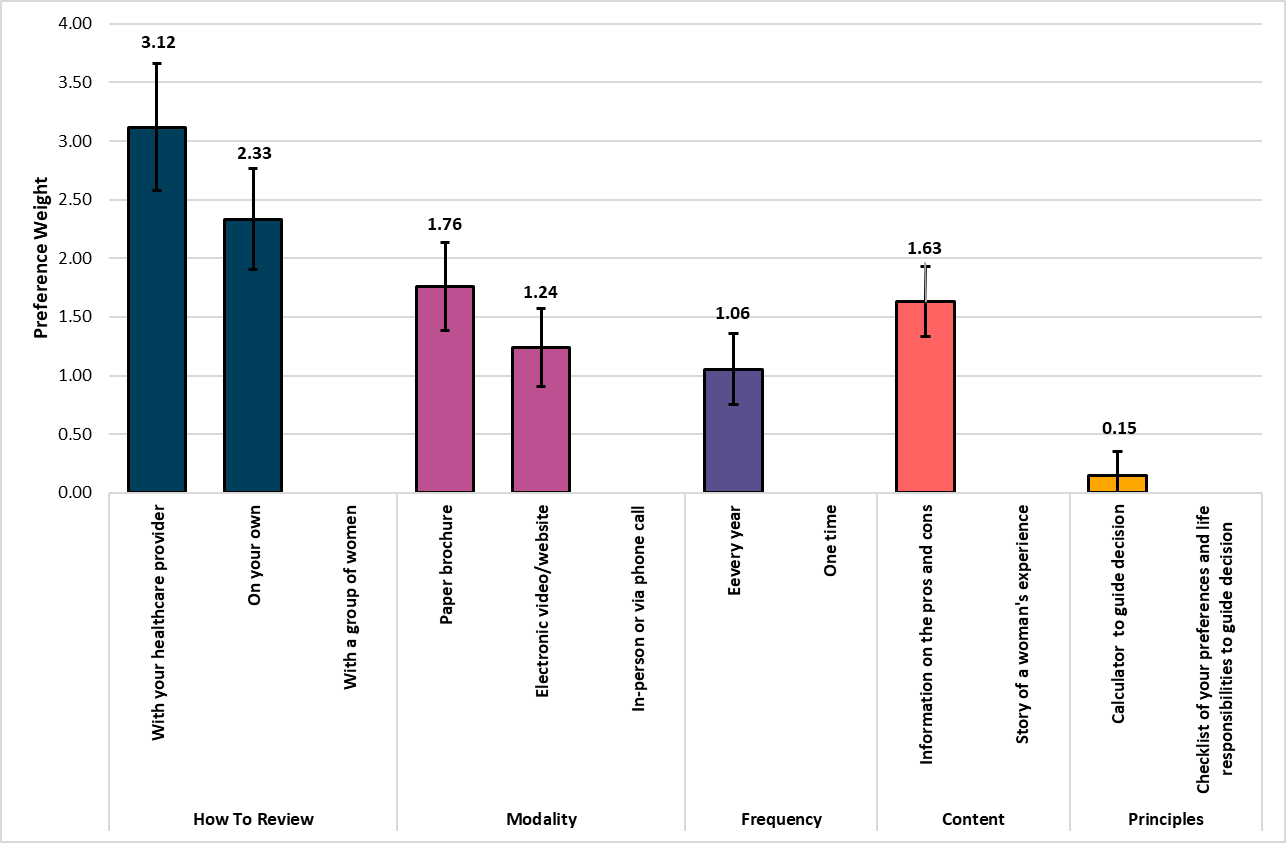


Source: 2024 De-implementation of Mammography Survey, *N* = 673.

Figure A-2. Random-Parameters Logit Preference Weights (Full Profile DCE Questions Only)


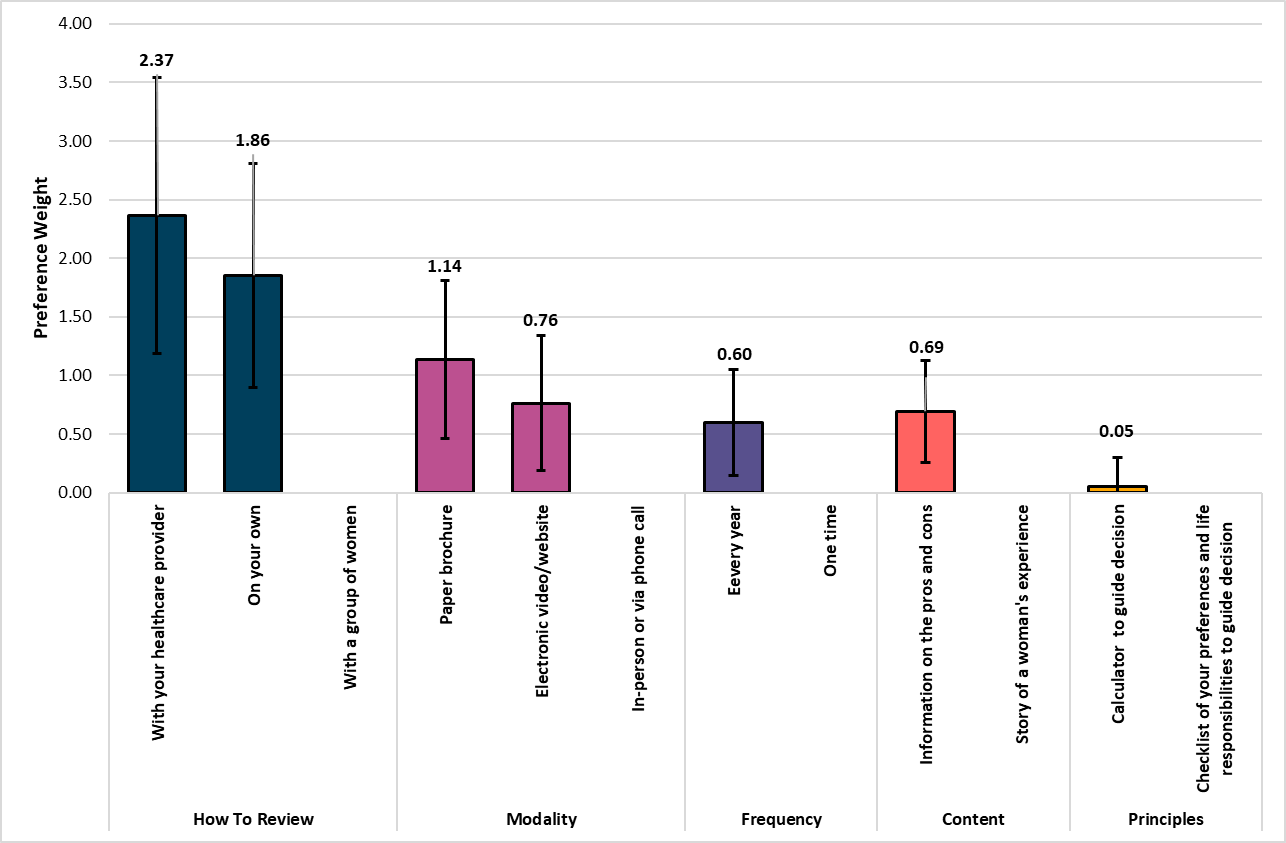


Source: 2024 De-implementation of Mammography Survey, *N* = 673.

Figure A-3. Random-Parameters Logit Preference Weights (All DCE Questions)


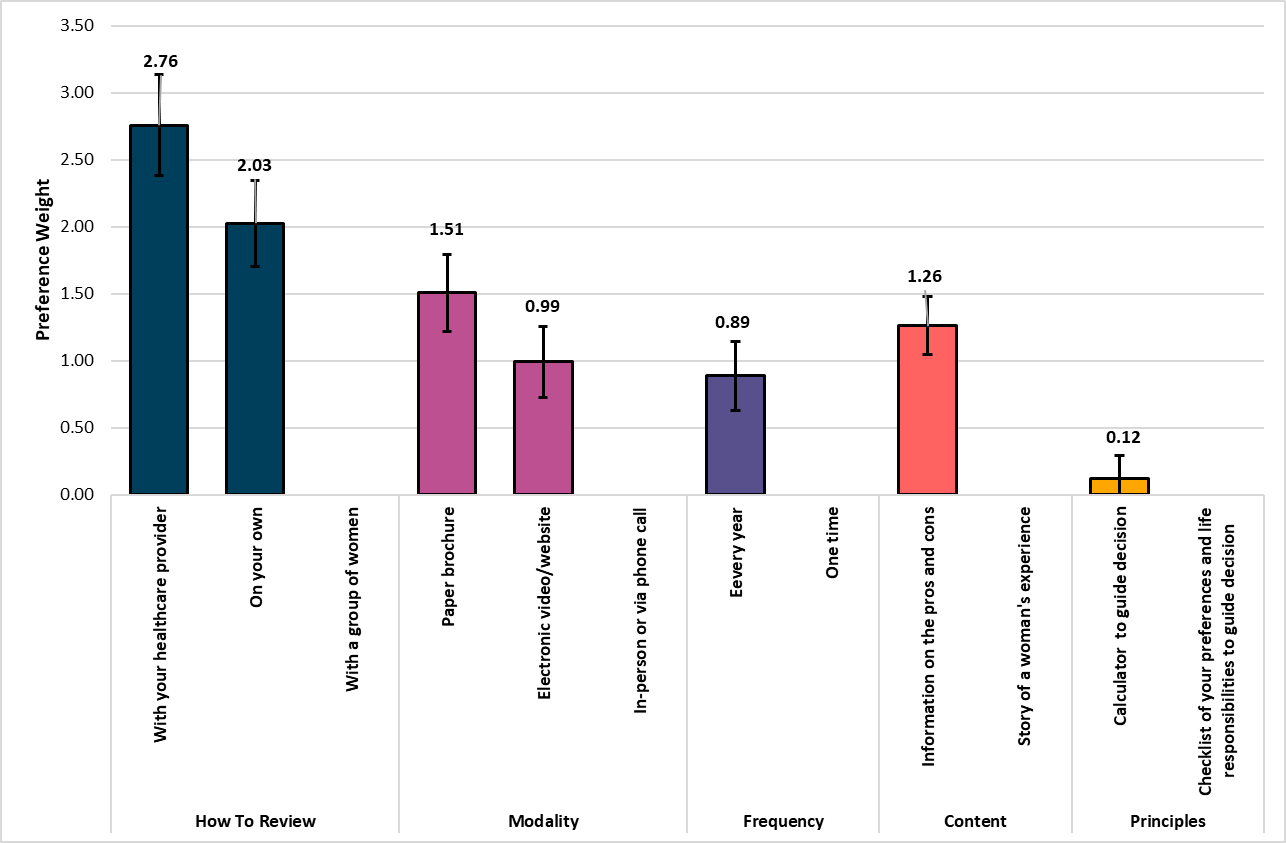


Source: 2024 De-implementation of Mammography Survey, *N* = 673.

Table A‑2. **Attribute Importance Score by RUM Model Estimated Using Random-Parameters Logit**

| Attribute | Partial Profile DCE Questions Only | Full Profile DCE Questions Only | All DCE Questions |
| --- | --- | --- | --- |
| How to review the information | 43.6 [39.4 - 47.8] | 47.9 [40 - 55.8] | 44.9 [41.2 - 48.6] |
| Modality of Information | 23.3 [18.8 - 27.8] | 23.0 [14.9 - 31.2] | 23.2 [19.1 - 27.3] |
| Frequency | 9.6 [6 - 13.2] | 12.9 [6.1 - 19.7] | 10.4 [6.7 - 14] |
| Education Content | 22.9 [19.7 - 26] | 15.2 [9.7 - 20.6] | 20.9 [18.2 - 23.7] |
| Guiding Principles to Inform the Content | 0.7 [-2.3 - 3.7] | 1.1 [-4.4 - 6.5] | 0.6 [-2.3 - 3.5] |

Source : 2024 De-implementation of Mammography Survey, *N* = 673.

Note: The 95% confidence interval (CI) is included in brackets. When the 95% CI around a mean importance score includes zero, the mean importance is not statistically different from zero. Also, if the 95% CIs around 2 different importance scores do not overlap, then the mean scores are statistically significantly different at the 5% level.

**Figure A-4. Attribute Importance Scores by RUM Model Estimated Using Random-Parameters Logit**

This figure represents attribute importance scores by RUM model estimated using random parameters logit. The vertical bars surrounding each mean preference weight denote the 95% confidence interval (CI) about the point estimate. If the CIs do not overlap for pairs of levels in a particular attribute, the mean estimates are statistically significantly different from each other at the 5% level of significance.


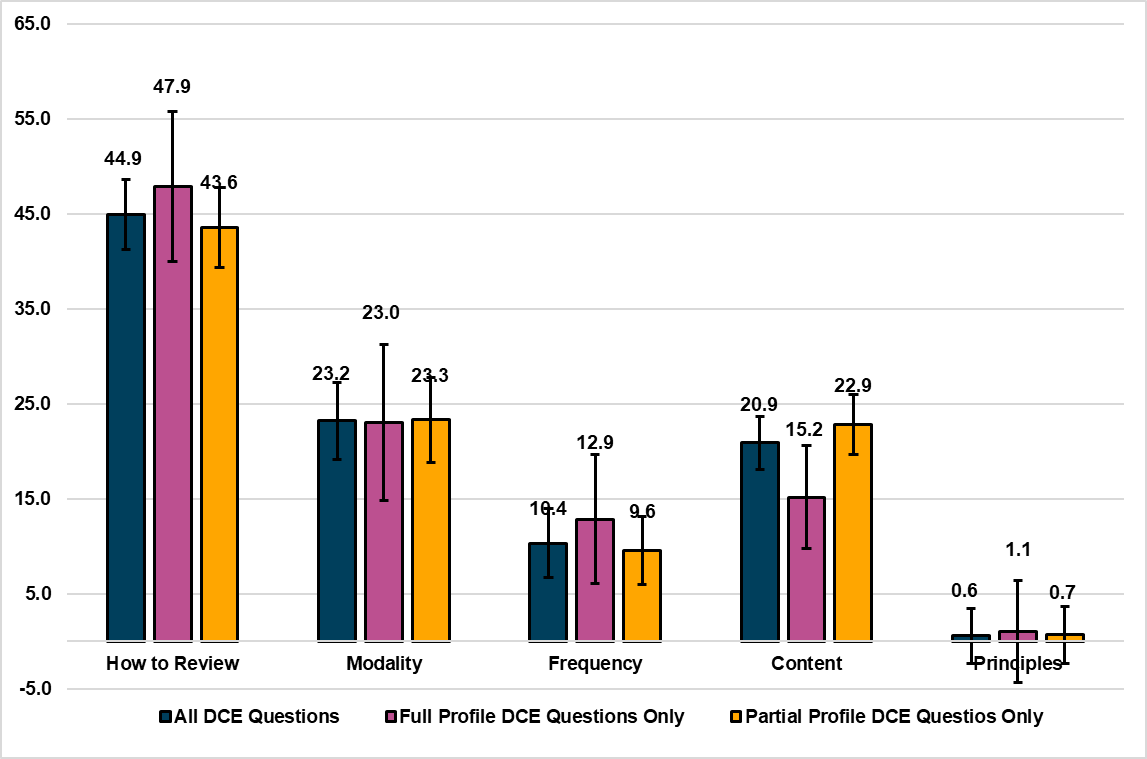


Source: 2024 De-implementation of Mammography Survey, *N* = 673.

Figure A-5. Attribute Importance Score Estimated Using Random-Parameters Logit (All DCE Questions)

This figure represents attribute importance scores estimated using all DCE questions. The vertical bars surrounding each mean preference weight denote the 95% confidence interval (CI) about the point estimate. If the CIs do not overlap for pairs of levels in a particular attribute, the mean estimates are statistically significantly different from each other at the 5% level of significance.


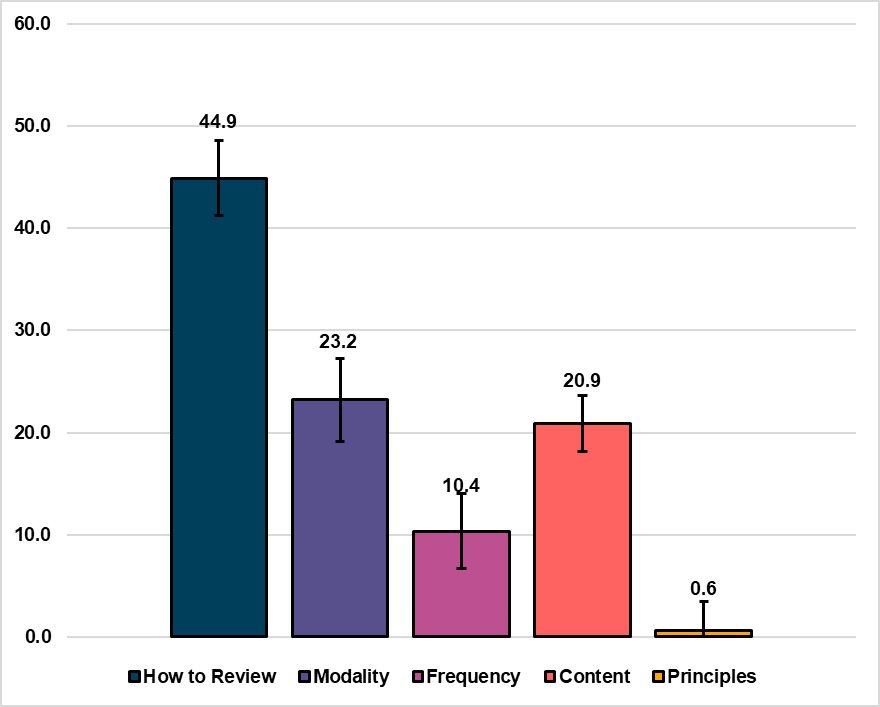


Source: 2024 De-implementation of Mammography Survey, *N* = 673.

# **3. References**

Gonzalez, J. M. (2019). A guide to measuring and interpreting attribute importance. *Patient, 12*(3), 287-295. <https://doi.org/10.1007/s40271-019-00360-3>

Hauber, A. B., Gonzalez, J. M., Groothuis-Oudshoorn, C. G., Prior, T., Marshall, D. A., Cunningham, C., MJ, I. J., & Bridges, J. F. (2016). Statistical Methods for the Analysis of Discrete Choice Experiments: A Report of the ISPOR Conjoint Analysis Good Research Practices Task Force. *Value in Health, 19*(4), 300-315. <https://doi.org/10.1016/j.jval.2016.04.004>

Hole, A. R. (2007). Fitting mixed logit models by using maximum simulated likelihood. *The Stata Journal, 7*(3), 388-401. <https://doi.org/10.1177/1536867x0700700306>

Janssen, E. M., Hauber, A. B., & Bridges, J. F. (2018). Conducting a discrete-choice experiment study following recommendations for good research practices: An application for eliciting patient preferences for diabetes treatments. *Value in Health, 21*(1), 59-68.

Johnson, F. R., Yang, J. C., & Reed, S. D. (2019). The internal validity of discrete choice experiment data: a testing tool for quantitative assessments. *Value in health*, *22*(2), 157-160.

McFadden, D., & Train, K. (2000). Mixed MNL models for discrete response. *Journal of Applied Economics, 15*(5), 447-470. <https://doi.org/10.1002/1099-1255(200009/10)15:5%3C447::AID-JAE570%3E3.0.CO;2-1>

Appendix A: Random-Parameters Logit Results

Table A-1. Random-Parameters **Logit Results (Weighted)**

|  | **Partial Profile DCE Questions Only** | | **Full Profile DCE Questions Only** | | **All DCE Questions** | |
| --- | --- | --- | --- | --- | --- | --- |
|  | **Mean  (Std. Err.)** | **Odds  Ratio** | **Mean  (Std. Err.)** | **Odds  Ratio** | **Mean  (Std. Err.)** | **Odds  Ratio** |
| How to Review the Information | | | | | | |
| Reviewed with your healthcare provider v. reviewed with a group of women | 3.12*†  (0.32) | 22.6 | 2.89 (2.51) | 18.0 | 2.77*† (0.22) | 15.9 |
| Reviewed on your own v. reviewed with a group of women | 2.41*†  (0.24) | 11.2 | 2.39 (2.14) | 10.9 | 2.17*† (0.19) | 8.7 |
| Modality of Information | | | | | | |
| Delivered through a paper brochure v. delivered in person or via phone call | 1.67*†  (0.20) | 5.3 | 1.39 (1.15) | 4.0 | 1.43*† (0.16) | 4.2 |
| Delivered through an electronic video/website v. delivered in person or via phone call | 1.24*† (0.18) | 3.4 | 1.09 (0.98) | 3.0 | 1.04*† (0.14) | 2.8 |
| How Often You Receive | | | | | | |
| Received every year v. received one time | 0.68* (0.15) | 2.0 | 0.78 (0.66) | 2.2 | 0.64* (0.13) | 1.9 |
| Education Content | | | | | | |
| Information on the pros and cons of stopping mammograms v. a story of a woman’s experience with stopping mammograms | 1.64* (0.16) | 5.1 | 0.92 (0.85) | 2.5 | 1.29* (0.12) | 3.6 |
| Guiding Principles to Inform the Content | | | | | | |
| Calculator based on your age and health v. checklist of your preferences and life responsibilities | 0.05 (0.11) | 1.1 | -0.06 (0.18) | 0.9 | 0.04 (0.09) | 1.0 |

Source: 2024 De-implementation of Mammography Survey, *N* = 673.

Notes: Standard errors are in parentheses. A star (*) denotes the coefficient is statistically different from zero at the 5% significant level. A cross (†) denotes that two coefficients for the same attribute are statistically different from each other at the 5% significance level.

Figure A-1. Random-Parameters Logit Preference Weights (Partial Profile DCE Questions Only)


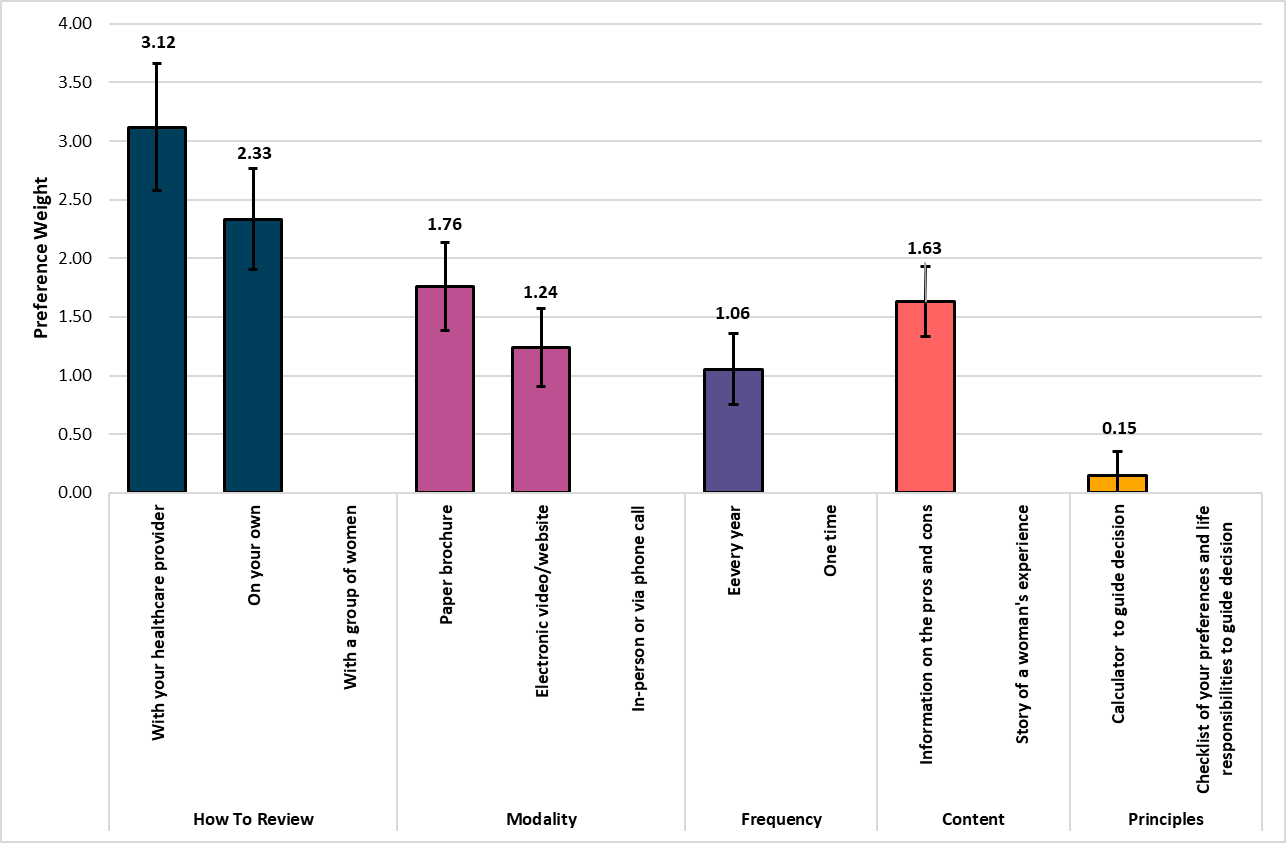


Source: 2024 De-implementation of Mammography Survey, *N* = 673.

Figure A-2. Random-Parameters Logit Preference Weights (Full Profile DCE Questions Only)


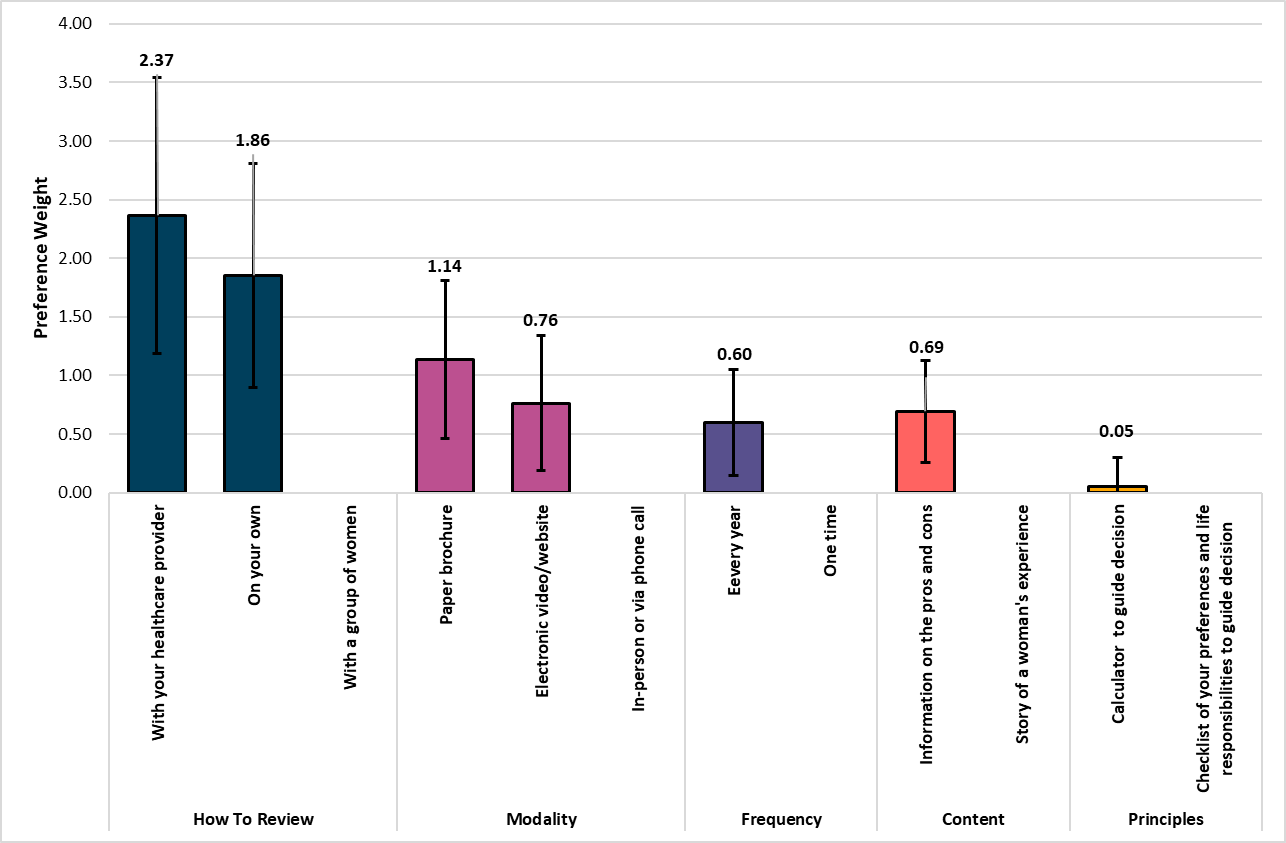


Source: 2024 De-implementation of Mammography Survey, *N* = 673.

Figure A-3. Random-Parameters Logit Preference Weights (All DCE Questions)


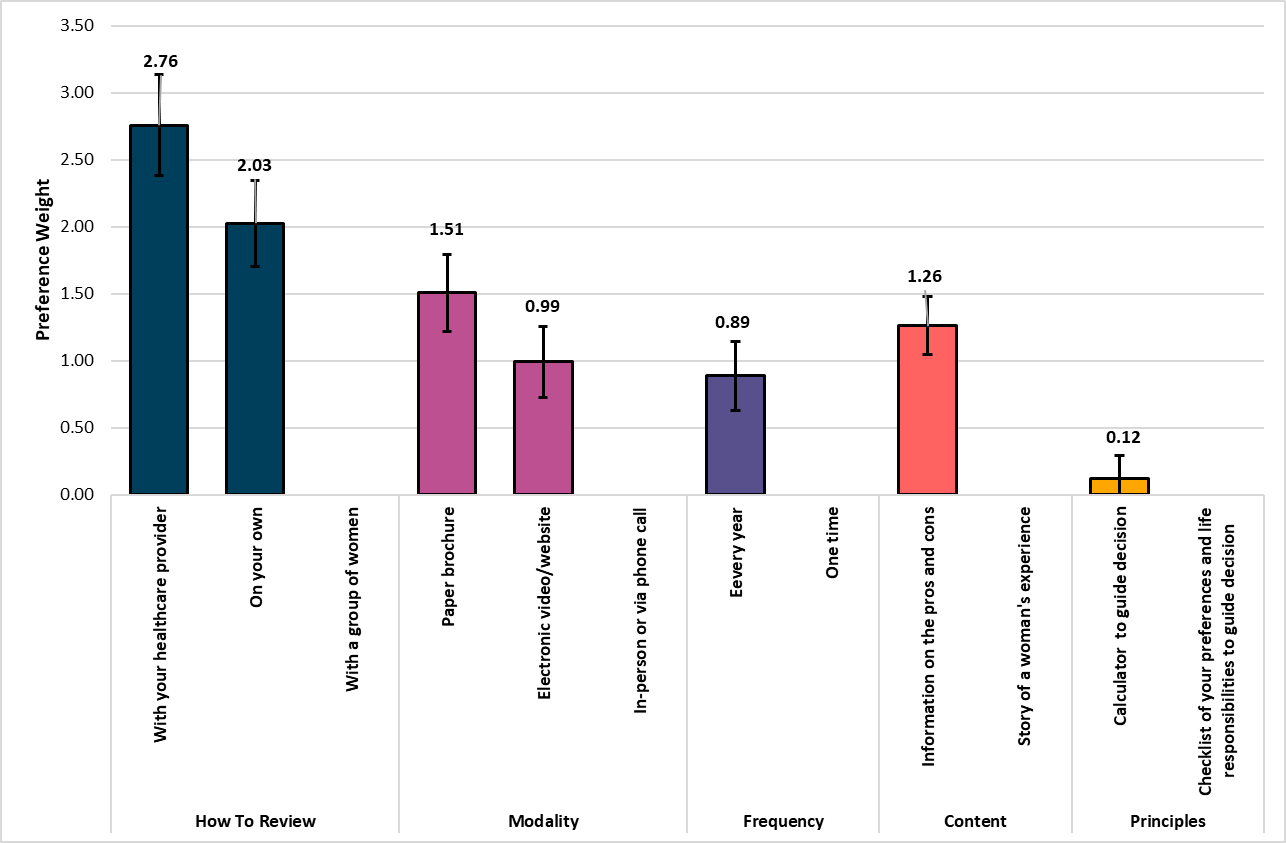


Source: 2024 De-implementation of Mammography Survey, *N* = 673.

Table A‑2. **Attribute Importance Score by RUM Model Estimated Using Random-Parameters Logit**

| Attribute | Partial Profile DCE Questions Only | Full Profile DCE Questions Only | All DCE Questions |
| --- | --- | --- | --- |
| How to review the information | 43.6 [39.4 - 47.8] | 47.9 [40 - 55.8] | 44.9 [41.2 - 48.6] |
| Modality of Information | 23.3 [18.8 - 27.8] | 23.0 [14.9 - 31.2] | 23.2 [19.1 - 27.3] |
| Frequency | 9.6 [6 - 13.2] | 12.9 [6.1 - 19.7] | 10.4 [6.7 - 14] |
| Education Content | 22.9 [19.7 - 26] | 15.2 [9.7 - 20.6] | 20.9 [18.2 - 23.7] |
| Guiding Principles to Inform the Content | 0.7 [-2.3 - 3.7] | 1.1 [-4.4 - 6.5] | 0.6 [-2.3 - 3.5] |

Source : 2024 De-implementation of Mammography Survey, *N* = 673.

Note: The 95% confidence interval (CI) is included in brackets. When the 95% CI around a mean importance score includes zero, the mean importance is not statistically different from zero. Also, if the 95% CIs around 2 different importance scores do not overlap, then the mean scores are statistically significantly different at the 5% level.

**Figure A-4. Attribute Importance Scores by RUM Model Estimated Using Random-Parameters Logit**

This figure represents attribute importance scores by RUM model estimated using random parameters logit. The vertical bars surrounding each mean preference weight denote the 95% confidence interval (CI) about the point estimate. If the CIs do not overlap for pairs of levels in a particular attribute, the mean estimates are statistically significantly different from each other at the 5% level of significance.


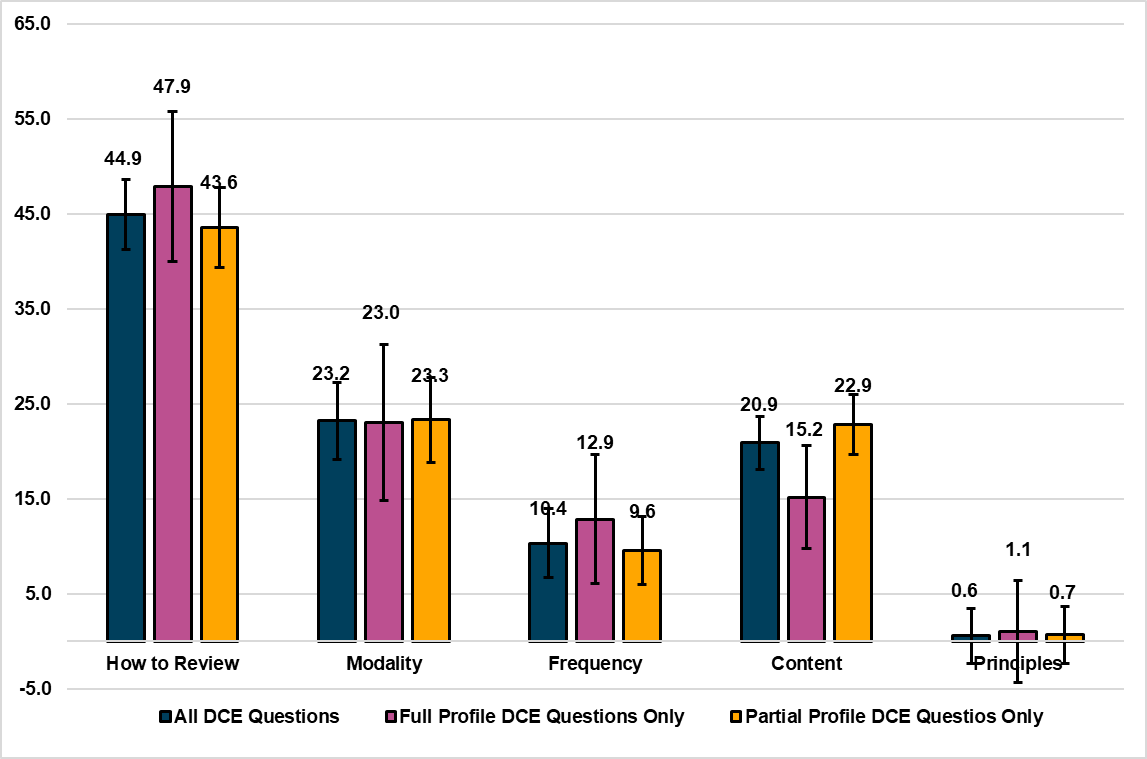


Source: 2024 De-implementation of Mammography Survey, *N* = 673.

Figure A-5. Attribute Importance Score Estimated Using Random-Parameters Logit (All DCE Questions)

This figure represents attribute importance scores estimated using all DCE questions. The vertical bars surrounding each mean preference weight denote the 95% confidence interval (CI) about the point estimate. If the CIs do not overlap for pairs of levels in a particular attribute, the mean estimates are statistically significantly different from each other at the 5% level of significance.


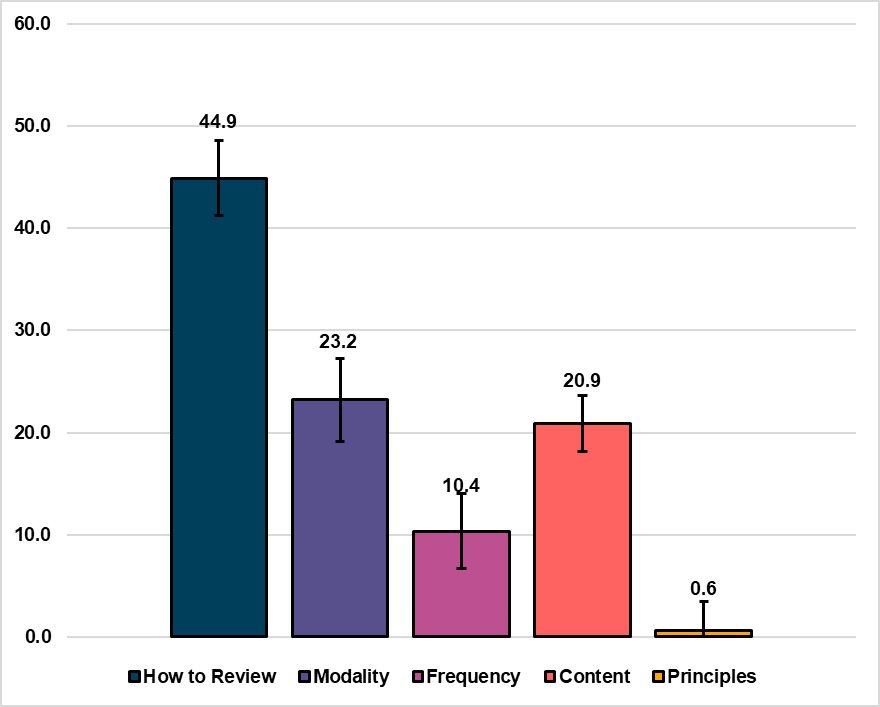


Source: 2024 De-implementation of Mammography Survey, *N* = 673.

Appendix B: NORC Survey Administered to Patients


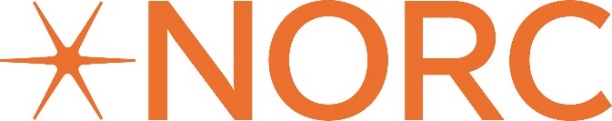

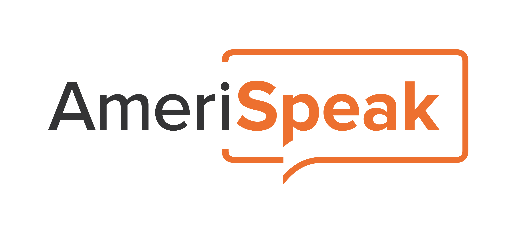


**Survey Questions***

**De-implementation of Mammography 2024**

Conducted for: Columbia University

Conducted by: NORC at the University of Chicago

Sample Source: AmeriSpeak Probability-Based Panel

Sampled Population: Women age 70+ who have never been diagnosed with cancer

Date Fielded: May 9^th^, 2024 – June 6^th^, 2024

|  |  |
| --- | --- |
|  |  |
|  |  |
|  |  |
|  |  |
|  |  |
|  |  |
|  |  |
|  |  |
|  |  |
|  |  |
|  |  |
|  |  |

**WINTRO_1.**

This survey is about women’s health.

## Part I – Mammogram Decisions

1. **In thinking about the next few years, would you say that you plan to get mammograms, you don’t plan to get mammograms, or you’re undecided?**
2. I plan to get a mammogram every year.
3. I plan to get a mammogram every 2 years or so.
4. I don’t plan to get a mammogram in the next few years.
5. I’ve decided to stop getting mammograms altogether.
6. I’m undecided.
7. Other:

1. **Has a healthcare provider ever told you that you could choose whether or not to have a mammogram?**
   1. Yes
2. No

#[SHOW IF Q2=1]

2a. **You said that a healthcare provider has told you that you could choose whether or not to have a mammogram. Please check all that apply:**

- - 1. My healthcare provider said I could stop getting mammograms altogether.
    2. My healthcare provider said I should continue to get mammograms.
    3. My healthcare provider said something else:

## Part II – About Breast Cancer Screening Recommendations and the Rethink Resource (DCE)

INTRODCE1.

**Please read each option carefully before selecting which one you prefer. Several options may seem repetitive. Reading the options carefully will help us avoid making incorrect conclusions. Your answers play a key role in making useful educational materials for patients like you.**

Which of the two options below is your preference for The Rethink Resource? Please select one.

Option A: _______ Option B: _______

**The Rethink Resource:**

is delivered through **an electronic video/website.**

**AND**

contains **a story of a woman’s experience with stopping mammograms**.

**The Rethink Resource:**

is delivered **through a paper brochure**.

**AND**

contains **information on the pros and cons of continuing to get mammograms.**

INTROQ3.

Thank you for answering the questions about your preferences regarding the Rethink Resource. We would like to learn more about your experience answering these questions.

**3a. Please indicate if you agree or disagree with the following statement:**

**“I found it easy to understand the questions above”.**

1. Strongly Agree
2. Agree
3. Neither Agree nor Disagree
4. Disagree
5. Strongly Disagree

**3b. Please indicate if you agree or disagree with the following statement:**

**“I found it easy to answer all the questions above”.**

1. Strongly Agree
2. Agree
3. Neither Agree nor Disagree
4. Disagree
5. Strongly Disagree

INTRODCE2.

Now, we’d like to ask three more questions about your preferences regarding the Rethink Resource using a wider combination of options.

**Please read each option carefully before selecting which one you prefer. Several options may seem repetitive. Reading the options carefully will help us avoid making incorrect conclusions. Your answers play a key role in making useful educational materials for patients like you.**

Which of the two options below is your preference for The Rethink Resource? Please select one.

Option A: _______ Option B: _______

**The Rethink Resource:**

is reviewed **with your healthcare provider**

**AND**

is delivered through an **electronic video/website**

**AND**

is **received every year**

**AND**

contains **information on the pros and cons of continuing to get mammograms**

**AND**

includes **a calculator based on your age and health to guide your decision on whether or not to stop mammograms.**

**The Rethink Resource:**

is reviewed **on your own**

**AND**

Is delivered through **a** **paper brochure**

**AND**

is **received one time**

**AND**

contains **a story of a woman’s experience with stopping mammograms**

**AND**

includes a **checklist of your preferences and life responsibilities to guide your decision on whether or not to stop mammograms**.

INTROQ4.

Thank you for answering the questions about your preferences regarding the Rethink Resource. We would like to learn more about your experience answering these questions.

**4a. Please indicate if you agree or disagree with the following statement:**

**“I found it easy to understand the questions above”.**

- - 1. Strongly Agree
    2. Agree
    3. Neither Agree nor Disagree
    4. Disagree
    5. Strongly Disagree

**4b. Please indicate if you agree or disagree with the following statement:**

**“I found it easy to answer all the questions above”.**

1. Strongly Agree
2. Agree
3. Neither Agree nor Disagree
4. Disagree
5. Strongly Disagree

**4c. We are asking this question just to check your attention. Please select “Blue” below.”**

1. Red
2. Blue
3. Green

## Part III: Communication and Decision-Making Preferences

INTROQ5.

Please tell us about your preferences for communication and decision-making around mammography.

**5. How would you prefer to receive The Rethink Resource? Select your top preference.**

1. In-person communication (e.g., with a healthcare provider)
2. Printed materials (brochures, information sheets, workbooks)
3. Electronic material (videos)
4. By phone
5. Through a Community Educator

#[SHOW IF Q5=2]

**5a. You said you would prefer to receive The Rethink Resource via printed materials (brochures, information sheets, workbooks). How would you like to receive these?**

1. Mailed to home
2. Given in waiting room
3. With mammogram result letters

#[SHOW IF Q5=3]

**5b. You said you would prefer to receive The Rethink Resource via electronic material (videos). How would you like to receive this?**

1. Mailed to home
2. Email
3. Text
4. Through an electronic medical record patient portal

**6. Many clinics send reminder letters for annual mammograms. Do you think clinics should stop sending mammogram reminder letters to older women?**

1. Yes
2. No
3. Not sure
4. **What is your preferred role in making decisions about mammography with a family member(s) and healthcare provider? Please select which one best describes your preferred role.**
5. I prefer to make the final decision about whether or not to get a mammogram.
6. I prefer a family member(s) make the final decision about whether or not I get a mammogram.
7. I prefer my healthcare provider make the final decision about whether or not I get a mammogram.
8. I prefer that my healthcare provider and I (but not a family member(s)) share responsibility for deciding whether or not getting a mammogram is right for me.
9. I prefer that my healthcare provider, a family member, and I share responsibility for deciding whether or not getting a mammogram is best for me.
10. I prefer that my healthcare provider and a family member(s) (but not I) share responsibility for deciding whether or not getting a mammogram is best for me.
11. I prefer that a family member(s) and I (but not my healthcare provider) share responsibility for deciding whether or not getting a mammogram is best for me.
12. **Mammograms have pros (benefits) and cons (possible risk). The benefits of mammograms include helping to catch breast cancer early. Some downsides of mammograms are unnecessary follow-up tests and procedures, possible costs, and treatments that might not help women live healthier or longer. As women get older, other health conditions may take priority, and getting more testing and treatment for breast cancer may not offer significant health benefits. The decision to stop getting mammograms after age 75 is a personal choice.**

**Having read the above, what do you think of the idea of stopping mammograms based on a woman’s age and health?**

1. Strongly Agree
2. Somewhat Agree
3. Neutral
4. Somewhat Disagree
5. Strongly Disagree

## Part IV - Medical History

Now, please answer a few questions about your health and medical history.

1. **Would you say your health in general is...?**
2. Excellent
3. Very good
4. Good
5. Fair
6. Poor
7. **Have you ever been diagnosed with breast cancer?**
8. Yes
9. No

77. Not sure

1. **Have any of your first-degree biological relatives (mother/father, sisters/brothers, or daughters/sons) been diagnosed with breast cancer? Please do not include adoptive or step relatives, but do include half-siblings.**
2. Yes
3. No

77. Not sure

#[SHOW IF Q12=1]

**12a. Who had breast cancer? Check all that apply**

1. My mother
2. One or more sisters
3. One or more daughters
4. A male relative
5. **When did you have your most recent mammogram to check for breast cancer?**
6. A year ago or less
7. More than 1 year, up to 2 years ago
8. More than 2 years, up to 3 years ago
9. More than 3 years ago
10. I’ve never had a mammogram

**Demographic Profile:**

**Additional questions asked of panelists prior to this survey**

**and are included with the survey data**

| Variable | Values |
| --- | --- |
| Gender | 1 = Male |
|  | 2 = Female |
| Age | Age in years |
| Age (7 categories) | 1 = 18-24; 2 = 25-34; 3 = 35-44; 4 = 45-54; 5 = 55-64; 6 = 65-74; 7 = 75+ |
| Age (4 categories) | 1 = 18-29; 2 = 30-44; 3 = 45-59; 4 = 60+ |
| Education (5 categories) | 1 = Less than HS |
|  | 2 = HS graduate |
|  | 3 = Some college/associates degree |
|  | 4 = Bachelor’s degree |
|  | 5 = Post grad study/professional degree |
| Race/Ethnicity | 1 = White, Non-Hispanic |
|  | 2 = Black, Non-Hispanic |
|  | 3 = Other, Non-Hispanic |
|  | 4 = Hispanic |
|  | 5 = 2+ races, Non-Hispanic |
|  | 6 = Asian/Pacific Islander, Non-Hispanic |
| Housing Type | 1 = A one-family house detached from any other house |
|  | 2 = A one-family house attached to one or more houses |
|  | 3 = A building with 2 or more apartments |
|  | 4 = A mobile home or trailer |
|  | 5 = Boat, RV, van, etc. |
| Household Income (18 categories) | 1 = Less than $5,000 2 = $5,000 to $9,999 |
|  | 3 = $10,000 to $14,999 4 = $15,000 to $19,999 |
|  | 5 = $20,000 to $24,999 6 = $25,000 to $29,999 |
|  | 7 = $30,000 to $34,999 8 = $35,000 to $39,999 |
|  | 9 = $40,000 to $49,999 10 = $50,000 to $59,999 |
|  | 11 = $60,000 to $74,999 12 = $75,000 to $84,999 |
|  | 13 = $85,000 to $99,999 14 = $100,000 to $124,999 |
|  | 15 = $125,000 to $149,999 16 = $150,000 to $174,999 |
|  | 17 = $175,000 to $199,999 18 = $200,000 or more |
| Household Income (9 categories) | 1 = Less than $10,000 |
|  | 2 = $10,000 to $19,999 |
|  | 3 = $20,000 to $29,999 |
|  | 4 = $30,000 to $39,999 |
|  | 5 = $40,000 to $49,999 |
|  | 6 = $50,000 to $74,999 |
|  | 7 = $75,000 to $99,999 |
|  | 8 = $100,000 to $149,999 |
|  | 9 = $150,000 or more |
| Household Income (4 categories) | 1 = Less than $30,000 |
|  | 2 = $30,000 to $59,999 |
|  | 3 = $60,000 to $99,999 |
|  | 4 = $100,000 or more |
| Marital Status | 1 = Married |
|  | 2 = Widowed |
|  | 3 = Divorced |
|  | 4 = Separated |
|  | 5 = Never married |
| Metropolitan Statistical Area  Status | 0 = Non-Metro |
|  | 1 = Metro (as defined US OMB Core-Based Statistical Area) |
| Home Internet Access | 0 = No |
|  | 1 = Yes |
| Telephone Service | 1 = Landline telephone only |
|  | 2 = Have a landline, but mostly use cellphone |
|  | 3 = Have cellphone, but mostly use landline |
|  | 4 = Cellphone only |
|  | 5 = No telephone service |
| Ownership of Living Quarters | 1 = Owned or being bought by you or someone in your household |
|  | 2 = Rented for cash |
|  | 3 = Occupied without payment of cash rent |
| Region 4 (US Census) | 1 = Northeast |
|  | 2 = Midwest |
|  | 3 = South |
|  | 4 = West |
| Region 9 (US Census) | 1 = New England |
|  | 2 = Mid-Atlantic |
|  | 3 = East-North Central |
|  | 4 = West-North Central |
|  | 5 = South Atlantic |
|  | 6 = East-South Central |
|  | 7 = West-South Central |
|  | 8 = Mountain |
|  | 9 = Pacific |
| State | State of residence |
| Household Size | Total number of members in household |
| HH members, age 0-1 | Known number of household members in age group |
| HH members, age 2-5 | Known number of household members in age group |
| HH members, age 6-12 | Known number of household members in age group |
| HH members, age 13-17 | Known number of household members in age group |
| HH members, age 18+ | Known number of household members in age group |
| Current Employment Status | 1 = Working - as a paid employee |
|  | 2 = Working - self-employed |
|  | 3 = Not working - on temporary layoff from a job |
|  | 4 = Not working - looking for work |
|  | 5 = Not working – retired |
|  | 6 = Not working – disabled |
|  | 7 = Not working – other |
